# Discrete and continuous character-based disparity analyses converge to the same macroevolutionary signal: a case study from captorhinids

**DOI:** 10.1038/s41598-017-17757-5

**Published:** 2017-12-13

**Authors:** Marco Romano, Neil Brocklehurst, Jörg Fröbisch

**Affiliations:** 1 Museum für Naturkunde, Leibniz-Institut für Evolutions- und Biodiversitätsforschung, Invalidenstr. 43, 10115 Berlin, Germany; 2 Dipartimento di Scienze della Terra, “Sapienza” Universita‘ di Roma, P.le A. Moro 5, 00185 Rome, Italy; 3 Institut für Biologie, Humboldt-Universität zu Berlin, Invalidenstr. 42, 10115 Berlin, Germany

## Abstract

The relationship between diversity and disparity during the evolutionary history of a clade provides unique insights into evolutionary radiations and the biological response to bottlenecks and to extinctions. Here we present the first comprehensive comparison of diversity and disparity of captorhinids, a group of basal amniotes that is important for understanding the early evolution of high-fiber herbivory. A new fully resolved phylogeny is presented, obtained by the inclusion of 31 morphometric characters. The new dataset is used to calculate diversity and disparity through the evolutionary history of the clade, using both discrete and continuous characters. Captorhinids do not show a decoupling between diversity and disparity, and are characterized by a rather symmetric disparity distribution, with a peak in occupied morphospace at about the midpoint of the clade’s evolutionary history (Kungurian). This peak represents a delayed adaptive radiation, identified by the first appearance of several high-fiber herbivores in the clade, along with numerous omnivorous taxa. The discrete characters and continuous morphometric characters indicate the same disparity trends. Therefore, we argue that in the absence of one of these two possible proxies, the disparity obtained from just one source can be considered robust and representative of a general disparity pattern.

## Introduction

Investigating the relationship between species richness and morphological disparity through deep time represents a popular approach towards revealing important evolutionary trends and highlighting biological signals of macroevolutionary radiations. In recent years such an approach has been applied to different groups among both vertebrates and invertebrates, including for example graptolites^1^, foraminifera^2^, arthropods^3^, ammonoids^4^, gastropods^5^, bivalves^6^, echinoderms^7^, trilobites^8^, fishes^9^, marine reptiles^10^, pterosaurs^11^, dinosaurs^12,13^, therapsids^14,15^, cetaceans^16^, and rodents^17^.

The close comparison of diversity (species richness) and disparity (morphological diversity) within a particular clade is a powerful tool to recognize possible extinction selectivity and evolutionary radiations, to test for macroevolutionary hypotheses and to study in detail possible morphological responses to ecological and environmental factors^7^. Such an approach, thus, allows the understanding of large-scale dynamics of biodiversity, framed within the context of evolutionary paleobiology.

The present contribution investigates the patterns of diversity and disparity, based on discrete characters on the one hand and continuous morphometric characters on the other hand, for the first time in captorhinids, a major group of late Paleozoic tetrapods. For several reasons captorhinids are central for understanding the early evolution of amniotes and tetrapods in general. First, they represent a speciose group of Paleozoic tetrapods with more than 25 currently recognized species and a long stratigraphic range spanning from the late Carboniferous up to the end of the Permian. The oldest taxon of the clade, *Euconcordia* (previously *Concordia*) is known from the Hamilton Quarry of Kansas, Virgilian in age^18^. Within the Permian, captorhinids reached a mostly cosmopolitan distribution, with representatives known from Africa, Europe, Asia, and North and South America^19^. Some of their features such as the swollen neural arches and heavily sculptured skull bones make the remains of the captorhinids very distinctive and easily recognizable^20^.

A crucial event in the evolutionary history of this group is the appearance of high-fiber herbivory, and related changes especially in the cranium and dentition at the transition from faunivorous to omnivorous taxa. Important evolutionary changes in this context include the transition from the classic single-row of recurved dentary and maxillary teeth, typical of insectivory, to bullet-shaped maxillary teeth, arranged in multiple rows (up to 11) in high-fiber herbivores. The ability to grind and shred the plant material during food processing is made possible by a propalinal motion of the lower jaw^20,21^.

Recently, Captorhinidae have been the subject of multiple macroevolutionary analyses. Brocklehurst^19^ quantitatively examined the connection between the acquisition of high-fiber herbivory and the evolution of body size in captorhinids, demonstrating a general decoupling of the two traits. In a second paper, based on discrete character changes across the phylogeny Brocklehurst^22^ examined the possible impact of diet evolution on rates of morphological change in Captorhinidae, showing that a significant increase in rates of evolution (concentrated in characters of the mandible and dentition) coincides with the transition to herbivory within this clade. In addition, herbivorous captorhinids display a greater morphological disparity (occupied morphospace) compared to that of faunivorous captorhinids, collectively indicating an adaptive radiation of the herbivorous members of the clade.

Currently, the most resolved and comprehensive phylogeny of captorhinids obtained by Liebrecht *et al*.^23^, and used by Brocklehurst^19,22^ for his evolutionary studies, shows an unresolved node at the base of the Captorhinidae, with *Euconcordia* (*Concordia* therein) and *Opisthodontosaurus* forming a basal polytomy. Thus, as a first step in the present contribution, a phylogenetic analysis of the group was conducted, for the first time including numerous additional morphometric characters both derived from the cranial and postcranial (appendicular) skeleton, which resulted in a completely resolved phylogeny of captorhinids. The new expanded dataset constitutes an exemplary case study to test the relationship between taxic diversity and morphological disparity in this successful clade of Paleozoic amniotes.

The questions that are addressed in this study include: i) Are diversity and disparity decoupled throughout the evolutionary history of captorhinids, as already found in several groups of both vertebrates and invertebrates? ii) Do the results obtained separately on the basis of the discrete and continuous morphometric characters indicate a significantly different evolutionary history, or do they converge to the same signal? iii) Is the trend of morphological disparity linked to the evolution of omnivory and herbivory within the clade, with the emergence of key innovations and new ecological niches? iv) Are captorhinids characterized by an early peak in diversity and disparity (i.e. bottom-heavy clade) as observed in other groups, followed by a relative stabilization or decrease in the number of taxa and occupied morphospace?

## Results

### Phylogenetic Analysis

First, a cladistic analysis was performed on the original dataset of Liebrecht *et al*.^23^ using the software PAUP* 4.0b10 for Windows^24^, and selecting the ‘protorothyridid’ eureptile *Protorothyris* as outgroup. The heuristic search algorithm was used with 1000 addition sequence replicates, to avoid the searches becoming trapped in a local tree-length minimum^25^. The parsimony analysis was identical to the results of Liebrecht *et al*.^23^ and found two equally parsimonious trees, 161 steps in length, with a consistency index (CI) of 0.578, homoplasy index (HI) of 0.422 and retention index (RI) of 0.743. The strict consensus tree (Appendix 1) shows a polytomy at the base of Captorhinidae; in the first topology, *Euconcordia* (previously *Concordia*) is the most basal taxon within Captorhinidae, whereas in the second tree *Euconcordia* and *Opisthodontosaurus* are joined in a sister group relationship at the base of Captorhinidae, followed by *Rhiodenticulatus* in an immediately more derived position.

**Figure 1:**
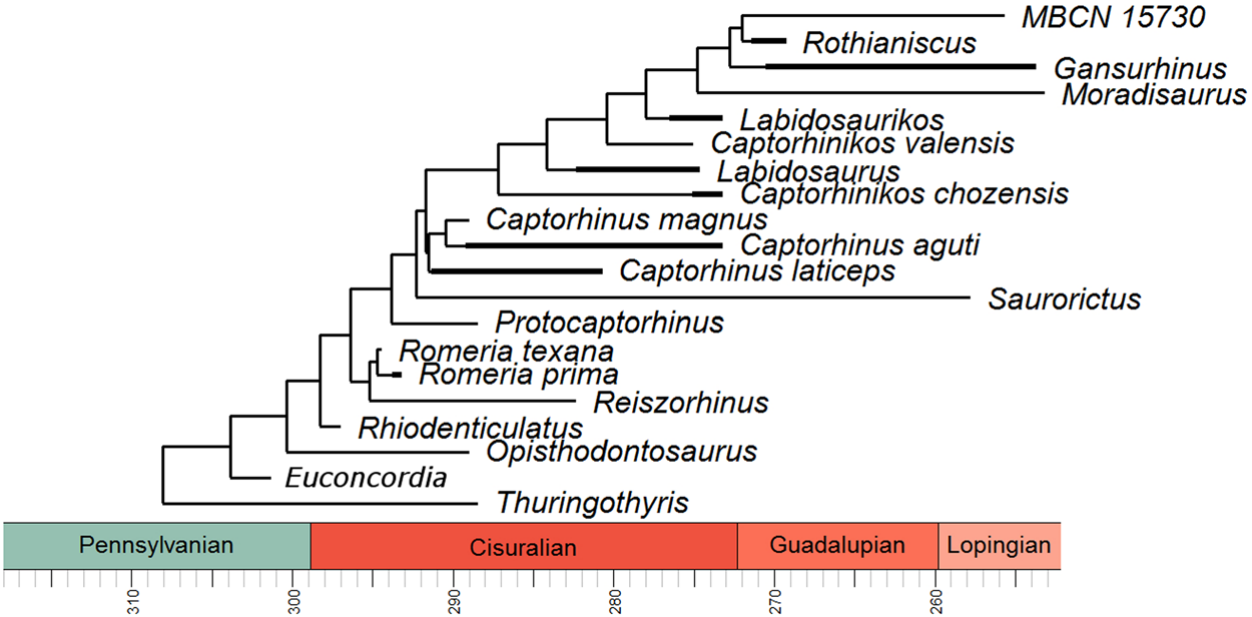
New fully resolved phylogeny of Captorhinidae.

To fully resolve the cladogram, a second cladistic analysis was performed on a new matrix that includes the 31 additional morphometric characters. The analysis conducted in the software TNT 1.5, which allows the insertion of continuous characters, found a single completely resolved tree (Fig. 1). The new cladogram confirms the first topology of the previous analysis, with *Euconcordia* representing the most basal taxon within Captorhinidae, followed by *Opisthodontosaurus* in an immediately more derived position. A support analysis using the Symmetric Resampling^26^ with 10.000 replicates (Appendix 1) shows that the node separating *Euconcordia* at the base of more derived captorhinids is well supported, being retained in the 72% of the replicates.

### Disparity and Diversity Analyses

**Figure 2:**
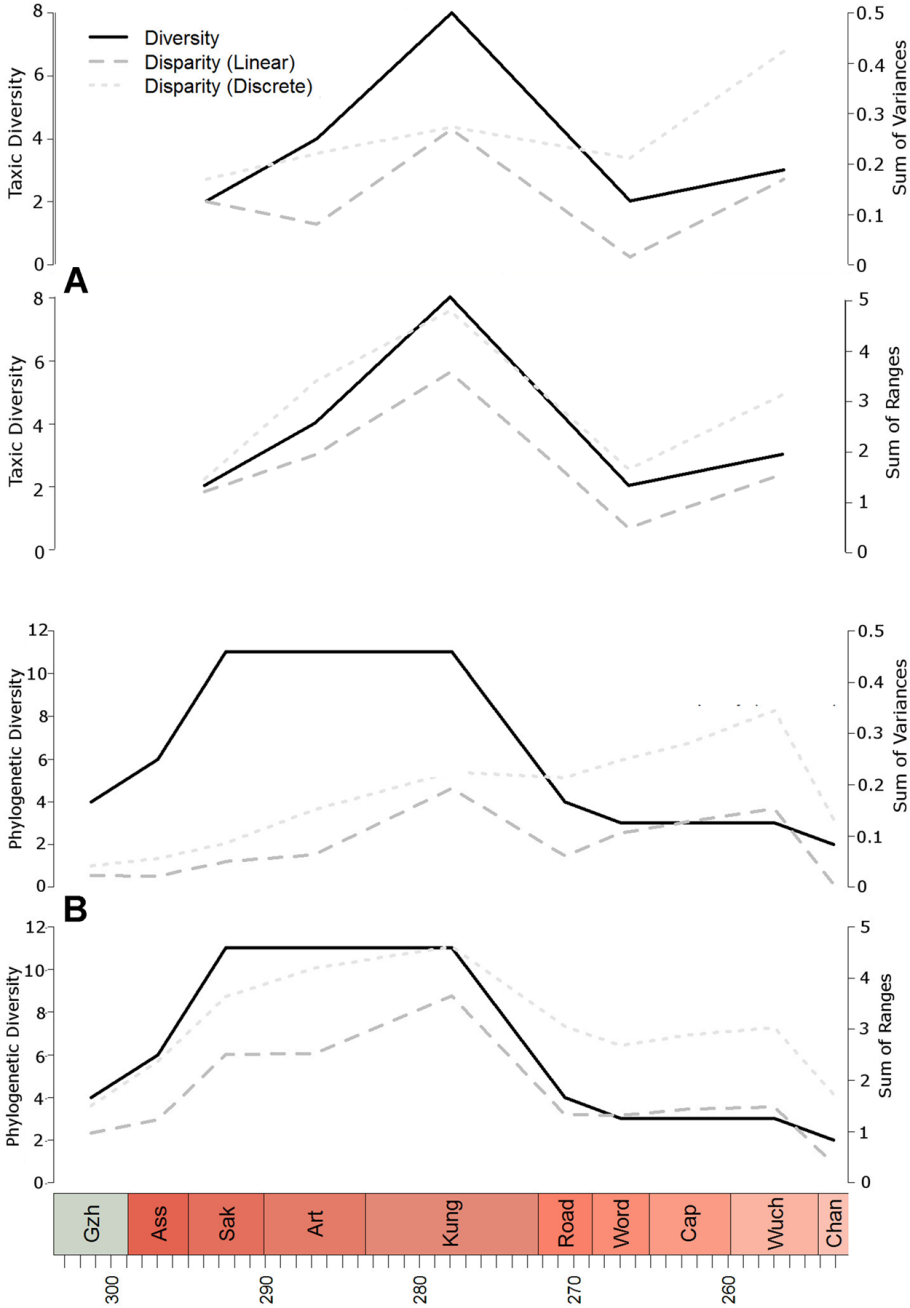
(**A**) Relationships of taxic diversity and morphological disparity, (**B**) relationships of phylogenetic diversity and morphological disparity incorporating ghost lineages over time. Disparity is expressed both as sum of variances and sum of ranges.

Figure 2 reflects the relationships of taxic diversity and morphological disparity over time, the latter expressed both as a sum of variances and sum of ranges. The sum of variances essentially measures the dispersal within morphospace of considered taxa^12,27–29^, whereas the sum of ranges can be considered as an indication of the amount of total morphospace occupation^12,30^. In addition, according to several studies^30–32^ the disparity measures based on variances are quite robust with respect to irregular sampling over time, whereas the metrics based on the sum of ranges are more consistent and robust with respect to taxonomic splitting and lumping^30,32^.

Most strikingly, the results in Fig. 2 show how both measures of disparity, sum of variances and sum of ranges, indicate the same trend and signal for the considered time bins. In addition, the disparity calculated separately on discrete and continuous morphometric characters are perfectly consistent. Equally striking is the very good concordance between the taxic diversity estimate and disparity in captorhinids through time. The diversity and disparity estimates not incorporating ghost lineages are relatively low early in the evolutionary history of the group, increase in the Artinskian, reach a maximum peak in the Kungurian (saturation both in terms of diversity and explored morphospace), decrease in the Guadalupian, and then rise again during the Lopingian (but without reaching the maximum diversity and disparity detected in the Kungurian).

The phylogenetic diversity estimate (species richness including ghost lineages) indicates an earlier increase in diversity (Fig. 2B), but also exhibits a decrease across the Kungurian/Roadian boundary. When ghost lineages are incorporated into disparity estimates, again there is a strong similarity between the disparity estimates inferred from discrete characters and those from continuous characters, and also between the disparity and diversity estimates. The exception is when considering the sum of variances with discrete characters, where the second, late Permian disparity peak is actually higher than the Kungurian peak.

The correspondence between the two disparity estimates is surprising since almost all of the principal coordinate scores inferred for each taxon from the discrete characters show very weak correlations with the respective principal component scores inferred from the continuous characters. This indicates that, although the two sets of morphological data are producing very different trait values for each species, the amount of variation inferred in each time bin does not vary substantially.

**Figure 3:**
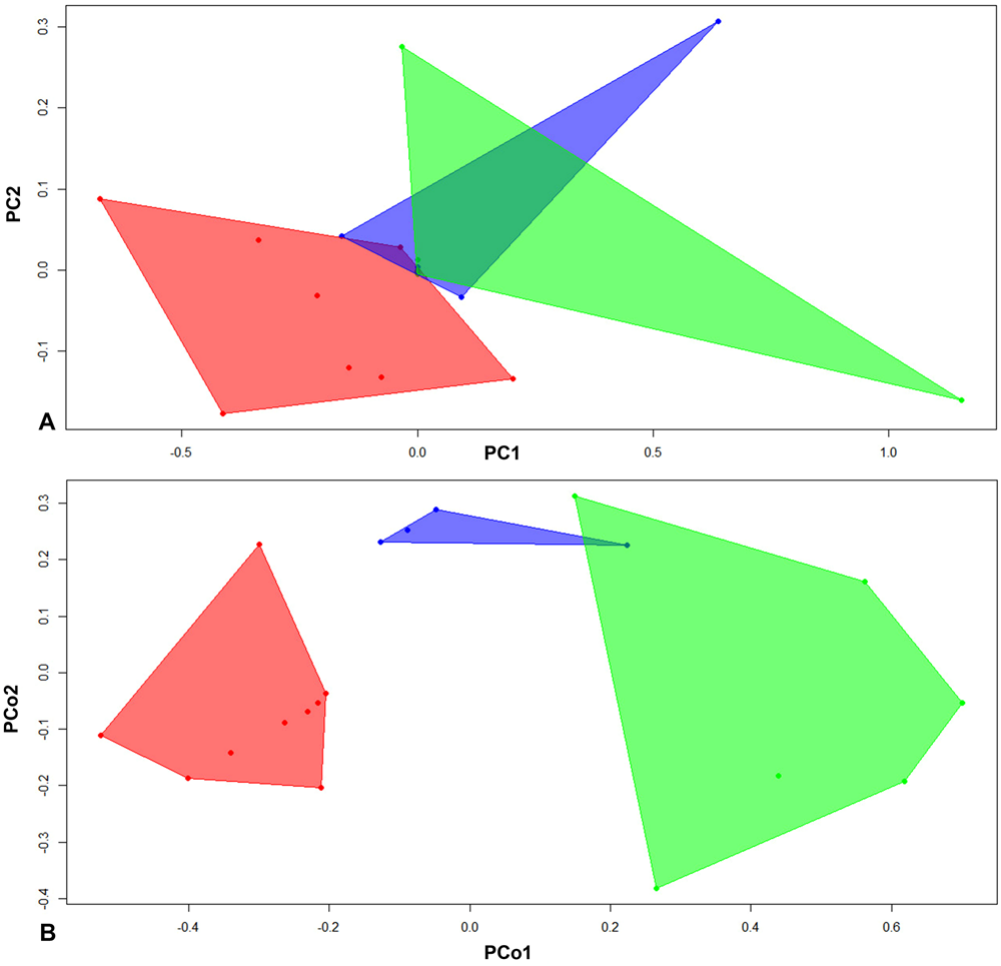
Scatter plots of principal components (based on continuous characters) and principal coordinates (based on discrete characters). **Red areas**: carnivores; **green areas**: herbivores; **blue areas**: omnivorous.

The scatter plots of the principal components (based on continuous characters) and the principal coordinates (based on discrete characters), shown in Fig. 3, provide valuable information regarding morphospace occupation. In both scatter plots, carnivores (red areas) and herbivores (green areas) form compact groups and occupy separate and well-defined portions of morphospace, with slight overlapping of convex hulls in continuous characters (Fig. 3A), whereas for discrete characters the two are almost completely separated (Fig. 3B). Another striking result is the distribution of omnivorous taxa in the two scatter plots (blue areas). In the case of discrete characters, the omnivores form a very compact small group in the morphospace, with little overlap with herbivores. In contrast, the scatter plot of principal components based on continuous characters indicates a distinct overlap of omnivores and herbivores, indicating that herbivores retained similar limb proportions to their omnivorous relatives, but this similarity in general *bauplan* is not recognizable in discrete characters intended to separate these taxa. Therefore, it is important to note that, even though discrete and continuous characters provide the same macroevolutionary signal for disparity through time, the two approaches display more divergent results in the morphospace analysis, depicted in the PCA and PCO graphs.

## Discussion

The consideration of continuous characters in phylogenetic analyses represents a topic of heated debates in the past decades. Some classic contributions, for example, question the validity and solidity of this ‘category’ of characters for reconstructing sound phylogenetic hypotheses^33–36^. On the other hand, several studies (some purely theoretical) fully support the use of continuous characters^37–40^, seeing no objective reason to reduce a feature that continuously varies among taxa into a few, arbitrary character states. On the empirical level, recent studies on both vertebrates and invertebrates^41–45^ have shown how the inclusion of substantial number of morphometric characters leads to completely resolved cladograms, useful then as basic evidence, for example, in evolutionary studies^46^.

In the present study, our phylogenetic analysis with the expanded character list, including 31 additional morphometric characters, has led to the most comprehensive fully resolved phylogeny for captorhinids, supporting the position of *Euconcordia* as the most basal member of Captorhinidae, as initially suggested by Müller and Reisz^18^ (*Concordia* therein). This new tree now forms the basis for subsequent macroevolutionary studies on this successful group of early amniotes.

The results of the diversity and disparity analyses of Captorhinidae indicate some general patterns. The trends of species richness and morphological disparity throughout the evolutionary history of the clade are generally consistent with one another. This contradicts numerous studies, including the seminal contribution by Foote^47^, conducted on several groups of organisms, which generally identify a decoupling of taxic diversity and morphological disparity. Case studies in this regard are available for example for graptolites^1^, anomodont therapsids^14^, trilobites^8^, and mysticetes^16^. In contrast, captorhinid diversity and disparity proceed largely in tandem, reaching a maximum peak during the Kungurian (Fig. 2). The exception (the sum of variances using discrete characters) appears to be driven by distribution of herbivores in their morphospace. The majority of the herbivore taxa are distributed towards the edge of the herbivore morphospace, with very few in the center. Thus, as taxa are removed from the morphospace through time by extinction, the size of the morphospace does not change substantially (as seen by the sum of ranges), but the lower number of data points relative to morphospace size means the variance does increase.

The pioneering work by Gould *et al*.^48^ already revealed a certain asymmetry in the macroevolutionary history of divergent taxonomic groups. According to Gould *et al*.^48^, clades can show maximum diversity in three different moments of their evolutionary history: close to the beginning (bottom-heavy clade), at approximately the midpoint (‘symmetrical’), or close to the end (top-heavy clade) of their stratigraphic range. Gould *et al*.^48^ recognized a predominance of bottom-heavy clades, with diversity that stabilizes or decreases over time. Higher early disparities have been recently supported empirically by Hughes *et al*.^49^, based on a meta-analysis of 98 vertebrate and invertebrate clades radiating throughout the Phanerozoic. Likewise, a bottom-heavy trend for disparity has been empirically found for example in blastozoans^50^, brachiopods^51–53^, Palaeozoic gastropods^54^, Neoproterozoic acritarchs^55^, crinoids^56–58^, echinoids^59^, anomodont therapsids^14^ and mysticetes^16^. A typical explanation for this general trend is essentially related to the “empty ecospace” model, where the colonization of new environments takes place as a result of free ecospace previously vacated by other occupants, or new resources become available by the acquisition of a single or series of novel “key” adaptations^49^. The clades, therefore, essentially enter new adaptive zones, experiencing rapid morphological evolution, then reaching a stasis when the ecological niches are mostly filled^8,9,60–62^.

In the case of the present study on captorhinids, we have not found the predominant early peak in disparity, which instead occurs at about the midpoint of the evolutionary history of the clade. All of the eight disparity curves exhibit a “center of gravity”^49^ with no significant deviation from the null expectation of 0.5, and all but one have a center of gravity between 0.4 and 0.6. This indicates symmetrical disparity profiles, with morphological diversity concentrated in the middle of the evolutionary history of the clade. Thus, the Kungurian peak can be interpreted as delayed or late saturation of morphospace by captorhinids. A similar late peak in disparity has, for example, also been found in blastoids^63^ and trilobites^64,65^ among invertebrates and pterosaurs among vertebrates^66^.

An interesting point to note is that the center of gravity of the four disparity profiles calculated from discrete characters are all higher (albeit by only a small amount) than the respective profiles calculated from continuous data. This would seem to suggest that the postcranial morphology (which dominates the continuous dataset) achieved its peak disparity earlier than the cranial morphology (which makes up considerably more of the discrete data). This supports the observation of Brocklehurst^22^, who noted that the carnivorous captorhinids (more diverse earlier in the clade’s evolutionary history) show higher rates of change in the postcranium than the later herbivores, whose morphological changes are concentrated in the dentition and mandible.

Instead, the late disparity peak and high “center of gravity” of the disparity profile of Captorhinidae might be said to better fit a diversity dependent model of diversification. It has been suggested that, under a “niche filling” paradigm, ecological opportunity should be the dominant factor influencing diversification rate, and therefore morphological evolution should be diversity-dependent, rather than time-dependent^67–69^. Thus, rates of morphological diversification should depend on lineage-diversity rather than clade-age, and the rates of diversification should decelerate as species diversity increases and the new region of ecospace is filled. In this context, the late origin of herbivory in captorhinids, combined with the reduction in species richness after the Kungurian, were crucial in driving the late disparity peak. Under a diversity dependence model, the new region of ecospace to be filled would reduce the “lineage density”^70^, allowing disparity to further rise throughout the Guadalupian and Lopingian.

**Figure 4:**
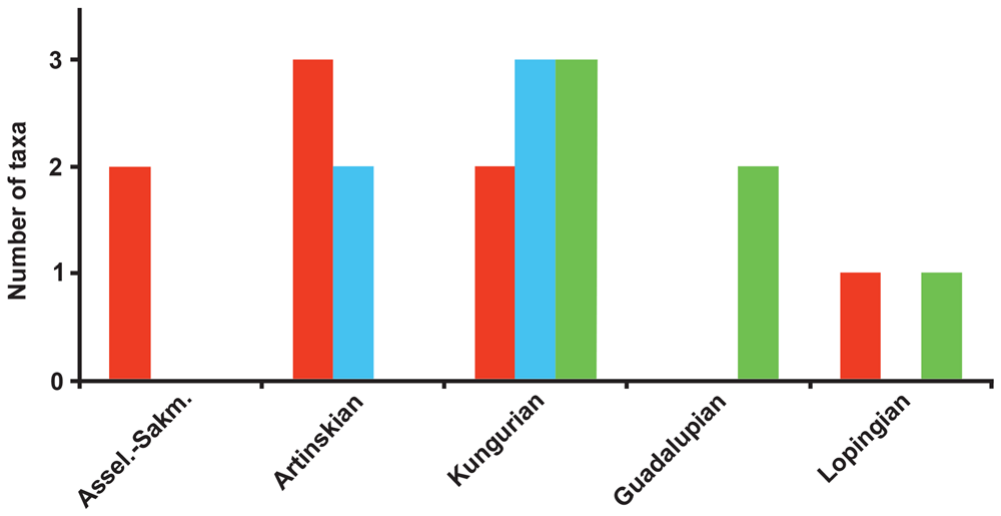
Number of taxa for each diet (carnivorous, omnivorous and herbivorous) for each time bin, derived from Brocklehurst (2017). **Red**: carnivores; **green**: herbivores; **blue**: omnivorous.

The number of taxa for each diet (carnivorous, omnivorous and herbivorous) for each time bin is provided in Fig. 4. The Asselian-Sakmarian interval is characterized only by carnivorous captorhinid taxa (i.e. *Rhiodenticulatus* and *Romeria prima*), the first omnivorous forms appear in the Artinskian (i.e. *Captorhinus aguti* and *C*. *magnus*), whereas the Kungurian peak is composed of higher numbers of omnivorous and herbivorous taxa (appearance of multiple tooth-rowed moradisaurines) and fewer carnivores. Thereafter, omnivorous forms disappear and captorhinid diversity is mostly driven by herbivores but also selected carnivorous taxa. Hence, the Kungurian disparity peak found in the present study is linked to the consistent presence of all three different diets. This is best interpreted as a delayed radiation, in this case connected to a fundamental key innovation: the appearance of high-fiber herbivory and of all the morphological and functional modifications, both in the cranial and postcranial skeleton, linked to the new diet. Our study therefore confirms the conclusion of Brocklehurst^22^ that the evolution of herbivory represents an adaptive radiation within Captorhinidae.

Strikingly, the here presented morphospace analyses reveal slightly divergent occupations by the three diets in the scatter plots of principal component and principle coordinate analyses (Fig. 3). In the PCA using the continuous characters, a consistent overlap of occupied morphospace between omnivorous and herbivorous taxa is detected, a signal not apparent in the PCo analysis based on discrete characters. This result is not surprising based on the discussion by Erwin^28^, according to which the potential morphospace derived from continuous characters should be larger compared to the one from discrete characters, even if considered for the same clade. Apparently discrete characters are empirically less suitable to detect general convergences, in our case study of captorhinids this applies to both the cranial and postcranial skeleton of omnivorous and herbivorous taxa.

To date, only few studies have investigated the patterns of disparity vs. diversity in early amniotes, of which the contemporaneous anomodont therapsids^14^ are particularly suitable for comparison. While, as discussed, captorhinids show a very low initial disparity coupled with low diversity, anomodonts were found to display a bottom-heavy disparity pattern with an early peak in the evolutionary history of the group, and thereafter gradually decreasing disparity^14^. A possible interpretation of this substantial difference between the two clades, is the different tempo and mode of diet and trophic niche exploration in the two groups. Anomodonts are exclusively represented by herbivorous taxa and the extensive exploration of the herbivorous niche at the beginning of their evolutionary history very likely led to an early morphospace saturation, then followed by stabilization or deceleration phenomena. In contrast, basal captorhinids were faunivorous and during the course of its evolutionary history the clade explored two additional trophic niches, i.e. omnivory and high-fiber herbivory. This likely resulted in a first increase in disparity during the Artinskian (appearance of omnivory) and a peak characterizing the Kungurian, with the appearance of several high-fiber herbivores. During the Kungurian peak all three explored diets were well represented, and the number of omnivorous and herbivorous taxa reached a maximum. In combination with the patterns seen in anomodont therapsids^14^, our results show how the evolution of novel trophic guilds within a clade can trigger a delayed adaptive radiation during its evolutionary history, leading also to a delayed saturation of occupied morphospace.

Finally, our study of captorhinid disparity shows that the two used proxies, discrete and continuous morphometric characters, generally converge on the same general trend through time in the evolutionary history of this clade of early amniotes. At least on a theoretical level, it has been hypothesized that these different approaches should capture different aspects of morphology, different scales of changes and also different degrees of redundancy^7^. In addition, the use of discrete characters could result in problems with character exhaustion, thus leading finally to an underestimation of disparity, when compared to results obtained from continuous characters^28^. However, a study of the radiation of the echinoid order Spatangoida empirically demonstrated that, independent of the choice of taxonomic rank (species or genera) and different disparity proxies such as landmarks, traditional morphometrics, and discrete characters, in the end all methods led to the same signal and general trend through time^7^. This was further supported in vertebrates by a study of pterosaur disparity, with different sources of morphological data providing congruent disparity results^66^.

Therefore, the present disparity study of another group of early amniotes represents a further empirical confirmation that different morphological proxies can lead to a generally consistent pattern throughout the evolutionary history of a group. This allows the important and encouraging conclusion that, even in the absence of other proxies, the disparity estimated from just one source can be considered robust and representative of a general disparity pattern.

## Material and Methods

### Phylogenetic Analysis

The most recent and comprehensive phylogenetic dataset of captorhinids from Liebrecht *et al*.^23^, was used as the basis for the current analysis. The taxa included in the analysis are the same considered by Liebrecht *et al*.^23^. In addition to the 75 original characters in Liebrecht *et al*.^23^, 31 morphometric characters were constructed and included in the matrix, of which 18 were based on the available postcranial (appendicular) and 13 on the cranial morphology. All measurements were taken from published material available in the literature. The full list of new characters, the bibliographic source for each considered element, and the anatomical measurements considered are given in Appendix 1 of the supplementary material.

The new characters, in the form of a-dimensional ratios, were included in the original matrix (Appendix 2) and a cladistic analysis was performed in TNT v.1.5^71^, which allows the use of continuous morphometric characters. The analysis was conducted using a traditional heuristic search and the New Technology search parameters incorporating the drift, fusion and sectorial algorithms, searching for the minimum tree length 100 times, which both led to the same final topology. To calculate the support of the individual nodes of the cladogram a Symmetric Resampling with 10,000 replicates was calculated, which avoids distortion of the actual group support (over- or underestimations) in cases when characters show different prior weights or state transformation costs are different^26^.

### Time Calibrating the Phylogeny

The most parsimonious phylogeny produced by the parsimony analysis was time calibrated using the method of Lloyd *et al*.^72^. This method was itself based on an approach by Hedman^73^, whereby the observed age of a node relative to its successive outgroups could be used to make inferences about sampling, and thereby assess how far back in time the node should be extended. Lloyd *et al*.^72^ modified this method to be applied to an entire phylogeny. The tree was time calibrated in R 3.3.2 (R Core Team 2016). In order to date the root node, stratigraphically consistent outgroups were required. In this case, the diapsid *Paleothyris* and the earliest known synapsid *Archaeothyris* were used. A maximum age constraint was placed on the root of 325 million years: the age of the origin of amniotes inferred by a recent molecular clock study^74^ 10,000 ages were produced for each node, and the time calibrated tree used in subsequent analyses was dated using the mean age of each node.

### Comparison of Disparity Metrics

Disparity of captorhinids through time was assessed using both the continuous and discrete characters. The continuous characters were subjected to a principal component analysis in the program PAST^75^ to derive a multivariate ordination space. The data, being ratios, were first subjected to the logratio transformation in order to correct for spurious correlations between the variables^44,75^. Missing data was treated with mean-value imputation. The discrete characters were used to generate a pairwise distance matrix, which was then subjected to a principal coordinate analysis. The distance matrix was generated using the R package Claddis^76^, using the Maximum Observable Rescaled distance metric, shown to perform better in datasets with large amounts of missing data^76^.

To assess the congruence between the patterns of morphological variation identified by the continuous and discrete characters, the correlation between the first 14 principal components and their respective principal coordinates was assessed using the Pearson correlation coefficient in R (only the first 14 were used as this was the number of principal coordinates exhibiting positive variance). Prior to carrying out the correlation tests, both datasets were transformed using phylogenetic independent contrasts^77^ in the R package ape^78^ to account for the phylogenetic non-independence of taxa. The correlations are shown in Table 1 of the Appendix 3 in supplementary material.

The captorhinid taxa were placed into five time bins, selected as containing two or more taxa and being of roughly equal length: Asselian-Sakmarian, Artinskian, Kungurian, Guadalupian, Lopingian. Four curves of disparity through time were calculated: sum of ranges and sum of variances, each calculated from the principal components and principal coordinates.

In order to account for the incompleteness of the fossil record, the four disparity curves were recalculated using the method proposed by Brocklehurst^22^. This method incorporates ghost lineages (lineages not sampled in a time bin but inferred to have been present from the phylogeny) into the disparity analysis, inferring the morphology of the unsampled lineages at a particular point in time assuming a Brownian motion model of trait evolution. This method was implemented in R using the script provided by Brocklehurst^22^. Since the incorporation of ghost lineages increases the sample size, a finer division of the time scale was possible, so the time bins used were the geological stages from the Gzhelian until the Changhsingian.

To identify whether morphological disparity was concentrated early or late in the clade’s history, all eight disparity curves (sum of variances and ranges, based on discrete and continuous characters, based on raw observations and incorporating information from the phylogeny) were assessed using the procedure of Hughes *et al*.^49^. The scaled center of gravity (CGS) of the disparity profiles was calculated, producing a value of between 0 and 1. A value closer to 0 indicates a “bottom-heavy” clade, with the disparity concentrated earlier in their history, while a value closer to 1 indicates a “top-heavy” clade, with the disparity concentrated later in the clade’s history. Taxon bootstrapping was used to assess significance of the deviation from 0.5, as well as to account for the fact that the null expectation will differ due to variation in the length of the time bins.

## Electronic supplementary material

Appendix 1

Appendix 2

Appendix 3
