# Supplementary material for: Discrete and continuous character-based disparity analyses converge to the same macroevolutionary signal: a case study from captorhinids
Source: Sci Rep. 2017 Dec 13;7:17531. doi: 10.1038/s41598-017-17757-5 (PMC5727480; doi:10.1038/s41598-017-17757-5)
Supplement: Supplementary file 1 — Appendix 1 [file 41598_2017_17757_MOESM1_ESM.pdf]

Supplementary information: Discrete and continuous character-based disparity analyses converge to the same macroevolutionary signal: a case study from captorhinids

Marco Romano[1,2,]* Neil Brocklehurst[1] and Jörg Fröbisch[1,3]

[1]Museum für Naturkunde, Leibniz-Institut für Evolutions- und Biodiversitätsforschung, Invalidenstr. 43, 10115 Berlin, Germany; [2]Dipartimento di Scienze della Terra, "Sapienza" Universita` di Roma, P.le A. Moro 5, 00185 Rome, Italy; [3]Institut für Biologie, Humboldt-Universität zu Berlin, Invalidenstr. 42, 10115 Berlin, Germany

Abstract

The relationship between diversity and disparity during the evolutionary history of a clade provides unique insights into evolutionary radiation and the biological response to bottlenecks and to extinctions. Here we present the first comprehensive comparison of diversity and disparity of captorhinids, a group of basal amniotes that is important for understanding the early evolution of high-fiber herbivory. A new fully resolved phylogeny is presented, obtained by the inclusion of 31 morphometric characters. The new dataset is used to calculate diversity and disparity through the evolutionary history of the clade, using both discrete and continuous characters. Captorhinids do not show a decoupling between diversity and disparity, and are characterized by a rather symmetric disparity distribution, with a peak in occupied morphospace at about the midpoint of the clade's evolutionary history (Kungurian). This peak represents a delayed adaptive radiation, identified by the first appearance of several high-fiber herbivores in the clade, along with numerous omnivorous taxa. The discrete characters and continuous morphometric characters indicate the same disparity trends. Therefore, we argue that in the absence of one of these two possible proxies, the disparity obtained from just one source can be considered robust and representative of a general disparity pattern.

Appendix 1

Source of measurements taken from the literature

Taxon	Femur	Tibia	Fibula	Humerus	Ulna	Radius	Skull
Protorothyris	X	X	X	X	X	X	Clark and Carrol, 1973
Paleothyris	Sumida, 1997	Carrol, 1969	Carrol, 1969	Sumida, 1997	X	X	Carrol, 1969
Thuringothyris	Müler et al., 2006	X	X	Muller et al., 2006	Muller et al., 2006	Muller et al., 2006	Boy and Martens, 1991
Euconcordia	X	X	X	X	X	X	Muller and Reisz, 2005
Romeria prima	X	X	X	X	X	X	Clark and Carrol, 1973
Romeria texana	X	X	X	X	X	X	Clark and Carrol, 1973
Protocaptorhinus	Olson, 1984	X	X	Olson, 1984	Olson, 1984	X	Olson, 1984
Rhiodenticulatus	X	X	X	X	X	X	X
Saurorictus	X	X	X	X	X	X	Modesto and Smith, 2001
Captorhinus laticeps	X	X	X	X	X	X	Heaton, 1979
Captorhinus aguti	Holmes, 2002	Holmes, 2002	Holmes, 2002	Holmes, 1977	Holmes, 1977	Holmes, 1977	Gaffney and Mc Kenna 1979
Captorhinus magnus	Kissel et al. 2002	X	X	X	X	X	X
Labidosaurus	Sumida, 1989	Sumida, 1989	Sumida, 1989	Sumida, 1989	Sumida, 1988	Sumida, 1989	Modesto et al., 2007
Labidosaurikos	X	X	X	X	X	X	Dodick and Modesto, 1995
Moradisaurus	O'Keef et al., 2005	O'Keef et al., 2005	O'Keef et al., 2005	X	X	X	X
Rothianiscus	Olson, 1965	X	X	X	Olson, 1965	X	X
Captorhinikos valensis	X	X	X	X	X	X	X
Gansurhinus	X	X	X	X	Reisz et al., 2011	X	X
Captorhinkos chozensis	Olson and Barghusen, 1982	Olson and Barghusen, 1982	Olson and Barghusen, 1982	Olson and Barghusen, 1962	Olson and Barghuse, 1982	Olson and Barghusen, 1982	Kuhn, 1969
Reiszorhinus	X	X	X	X	X	X	Sumida et al., 2010
Opisthodontosaurus	Reisz et al., 2015	X	X	Reisz et al., 2015	Reisz et al., 2015	Reisz et al., 2015	Reisz et al., 2015

New morphometric characters

Homologues points chosen for measurements in all long bones: **pw**, proximal width; **dw**, distal width; **n**, narrowing at mid-shaft; **tl**, bone total length.

Femur

Character 76: pw/tl

Character 77: dw/tl

Character 78: n/tl

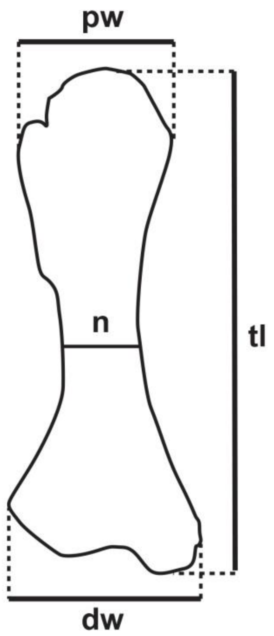

Tibia

Character 79: pw/tl

Character 80: dw/tl

Character 81: n/tl

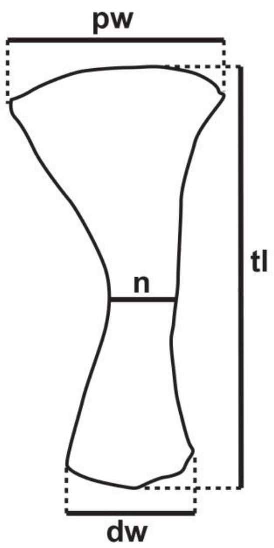

Fibula

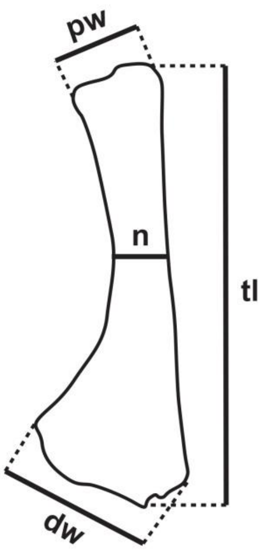

Character 82: pw/tl

Character 83: dw/tl

Character 84: n/tl

Humerus

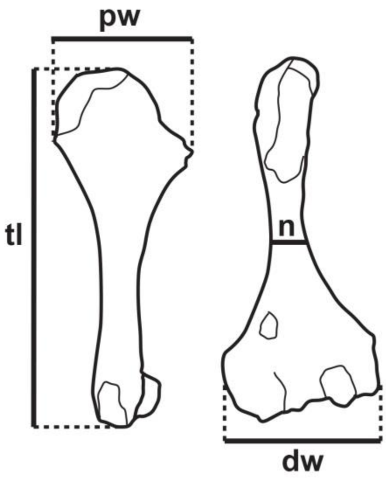

Character 85: pw/tl

Character 86: dw/tl

Character 87: n/tl

Ulna

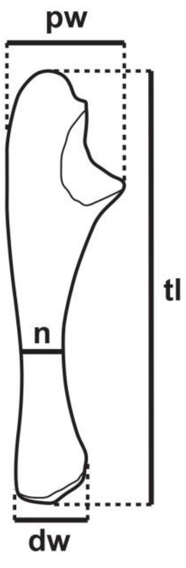

Character 88: pw/tl

Character 89: dw/tl

Character 90: n/tl

Radius

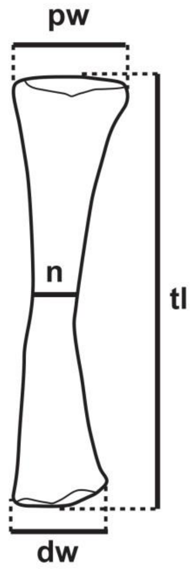

Character 91: pw/tl

Character 92: dw/tl

Character 93: n/tl

Cranial characters

Character 94: pol/tl

Character 95: aol/tl

Character 96: pw/tl

Character 97: pow/tl

Character 98: aow/tl

Character 99: fnw/tl

Character 100: pl/tl

Character 101: fl/tl

Character 102: nl/tl

Character 103: ppw/tl

Character 104: pfw/tl

Character 105: pnw/tl

Character 106: sw/tl

Character 107: fw/ol

Character 108: pl/ol

Character 109: sl/ol

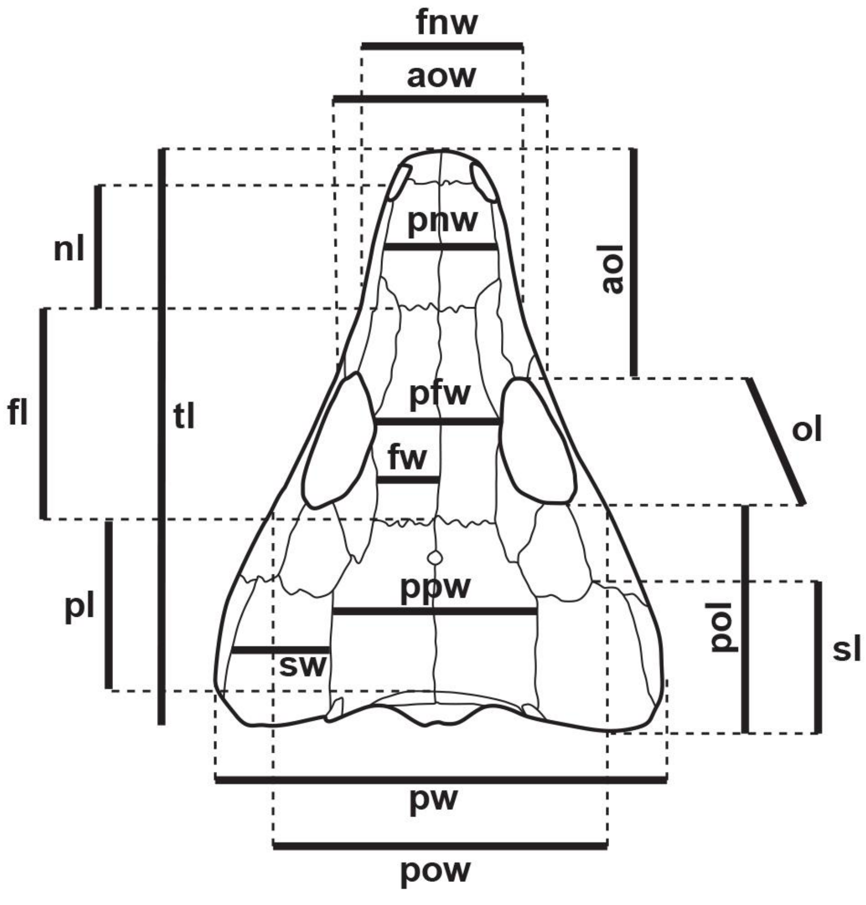

Homologues points chosen for measurements in the skull: tl, total length; **pol**, postero-orbital length; **aol**, antero-orbital length; **pw**, posterior width; **pow**, postero-orbital width; **aow**, antero-orbital width; **fnw**, width at the frontal-nasal suture; **pl**, parietal length; **fl**, frontal length; **nl**, nasal length; **ppw**, paried parietals width; **pfw**, paired frontals width; **pnw**, paired nasals width; squamosal width; **ol**, orbit length; **fw**, single frontal width.

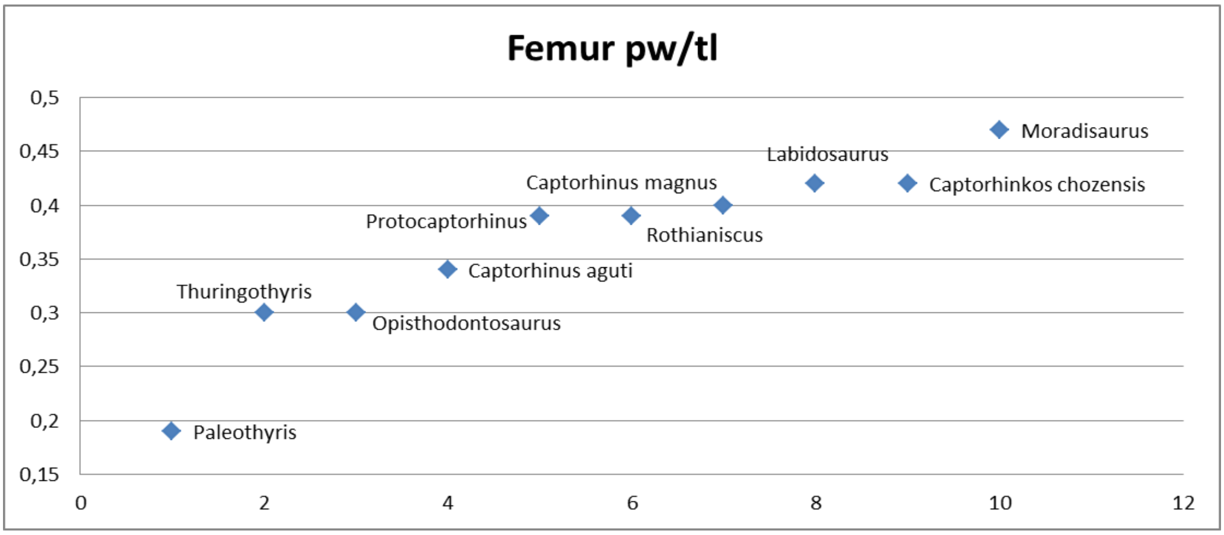
Femur pw/tl
0,5
0,45
0,4
0,35
0,3
0,25
0,2
0,15
0
2
4
6
8
10
12
Moradisaurus
Labidosaurus
Captorhinkos chozensis
Captorhinus magnus
Protocaptorhinus
Rothianiscus
Captorhinus aguti
Thuringothyris
Opisthodontosaurus
Paleothyris

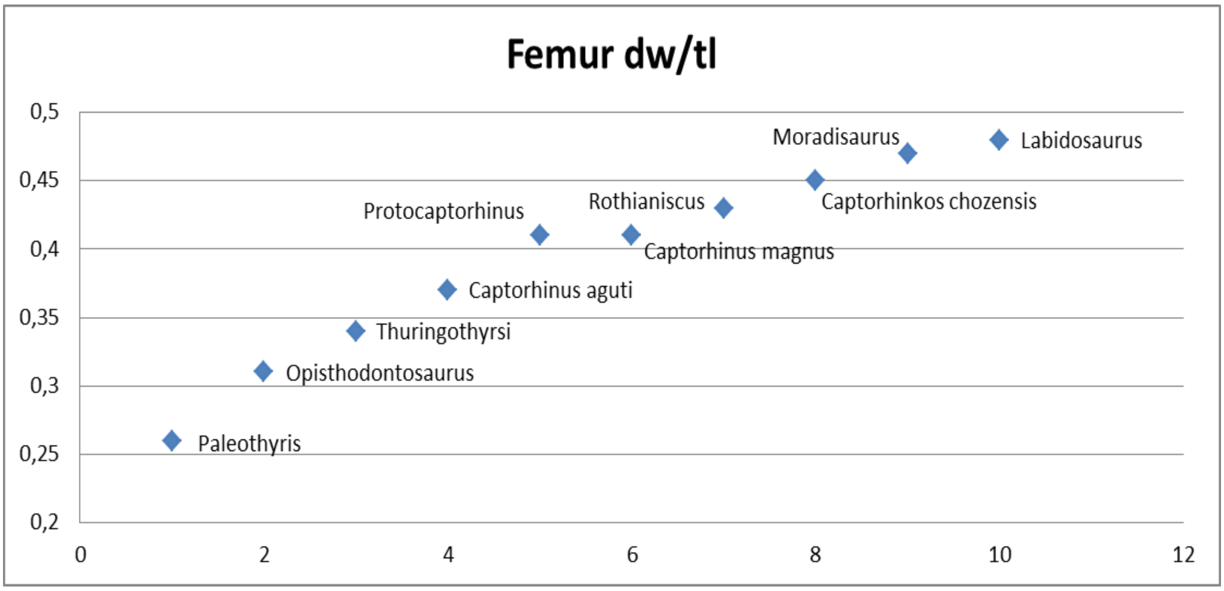
Femur dw/tl
0,5
0,45
0,4
0,35
0,3
0,25
0,2
0
2
4
6
8
10
12
Moradisaurus
Labidosaurus
Protocaptorhinus
Rothianiscus
Captorhinkos chozensis
Captorhinus magnus
Captorhinus aguti
Thuringothyrsi
Opisthodontosaurus
Paleothyris

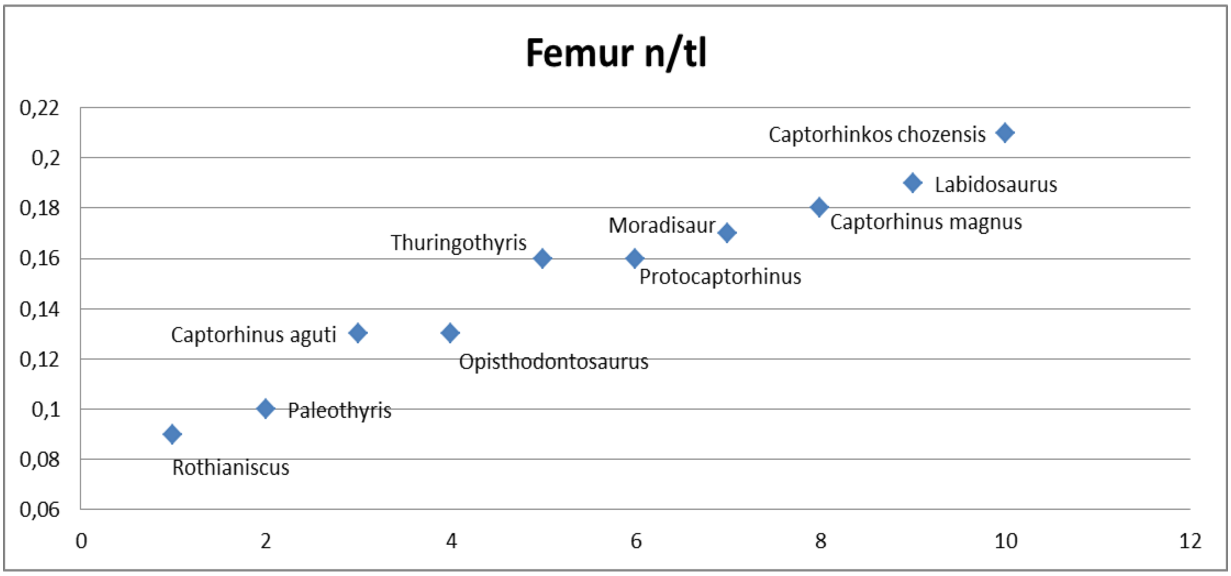
Femur n/tl
0,22
0,2
0,18
0,16
0,14
0,12
0,1
0,08
0,06
0
2
4
6
8
10
12
Captorhinkos chozensis
Labidosaurus
Moradisaur
Captorhinus magnus
Thuringothyris
Protocaptorhinus
Captorhinus aguti
Opisthodontosaurus
Paleothyris
Rothianiscus

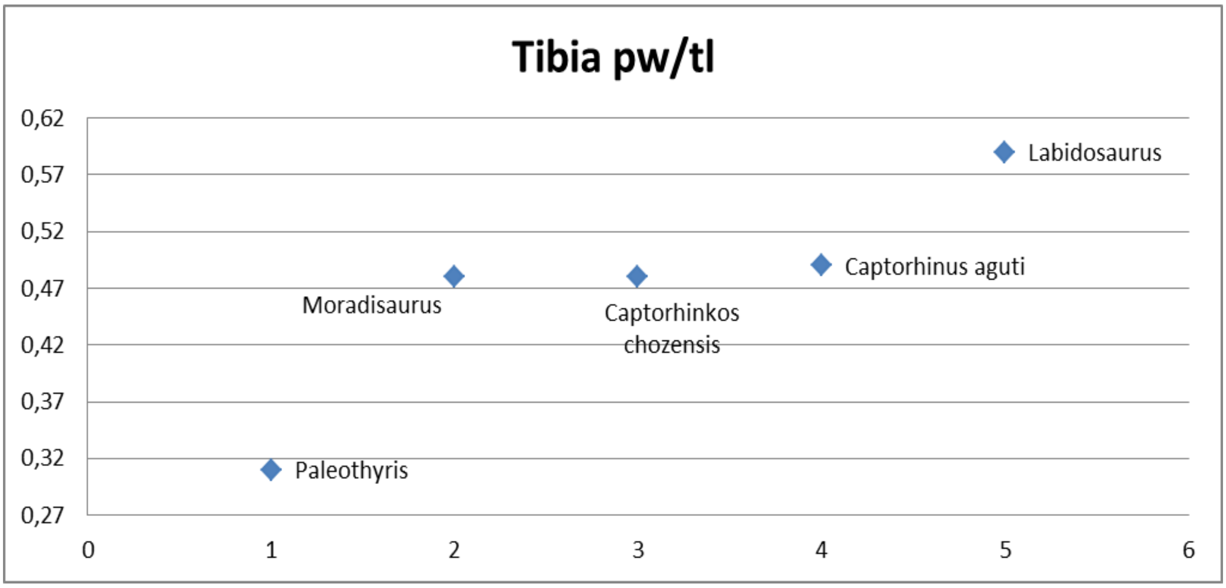
Tibia pw/tl
0,62
0,57
0,52
0,47
0,42
0,37
0,32
0,27
0
1
2
3
4
5
6
Labidosaurus
Captorhinus aguti
Moradisaurus
Captorhinkos chozensis
Paleothyris

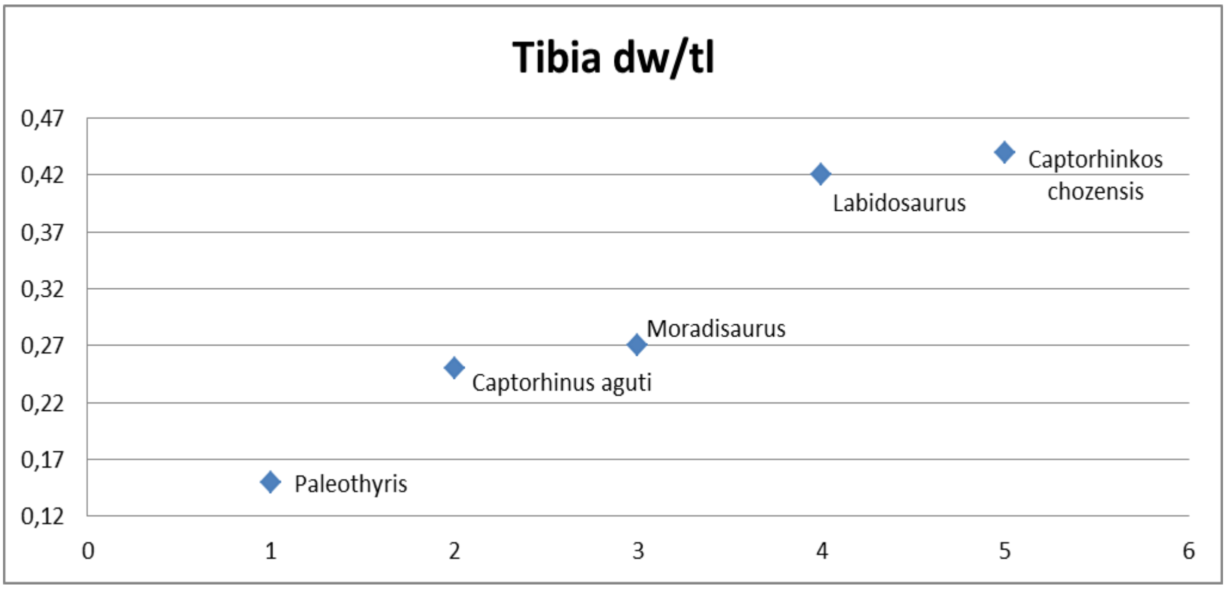
Tibia dw/tl
0,47
0,42
0,37
0,32
0,27
0,22
0,17
0,12
0
1
2
3
4
5
6
Captorhinkos chozensis
Labidosaurus
Moradisaurus
Captorhinus aguti
Paleothyris

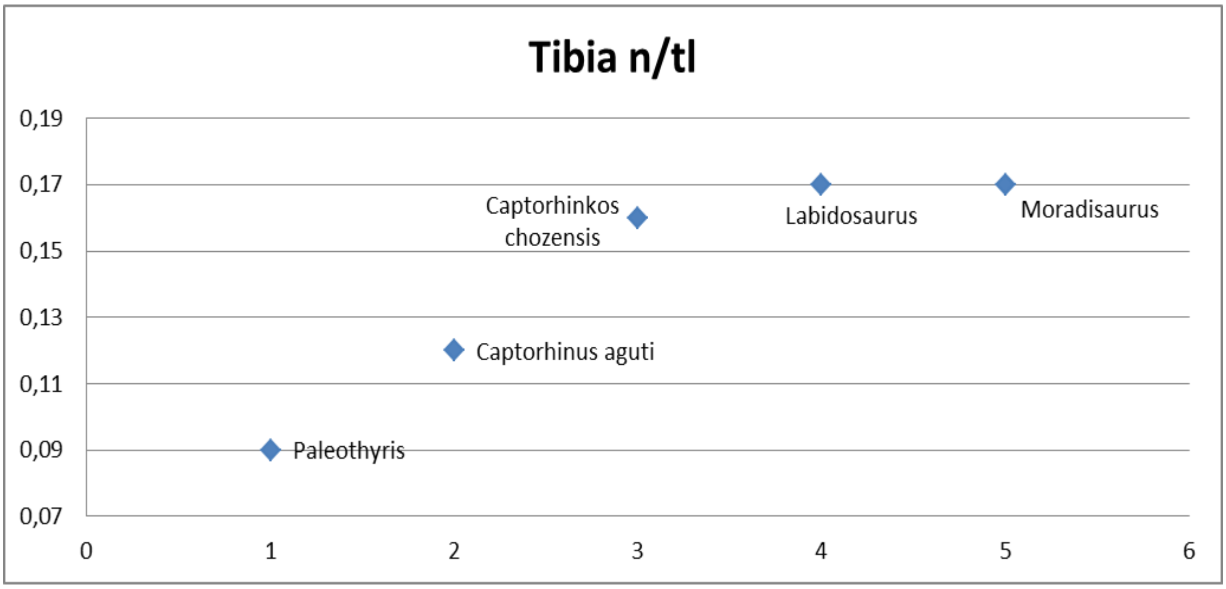
Tibia n/tl
0,19
0,17
0,15
0,13
0,11
0,09
0,07
0
1
2
3
4
5
6
Captorhinkos chozensis
Labidosaurus
Moradisaurus
Captorhinus aguti
Paleothyris

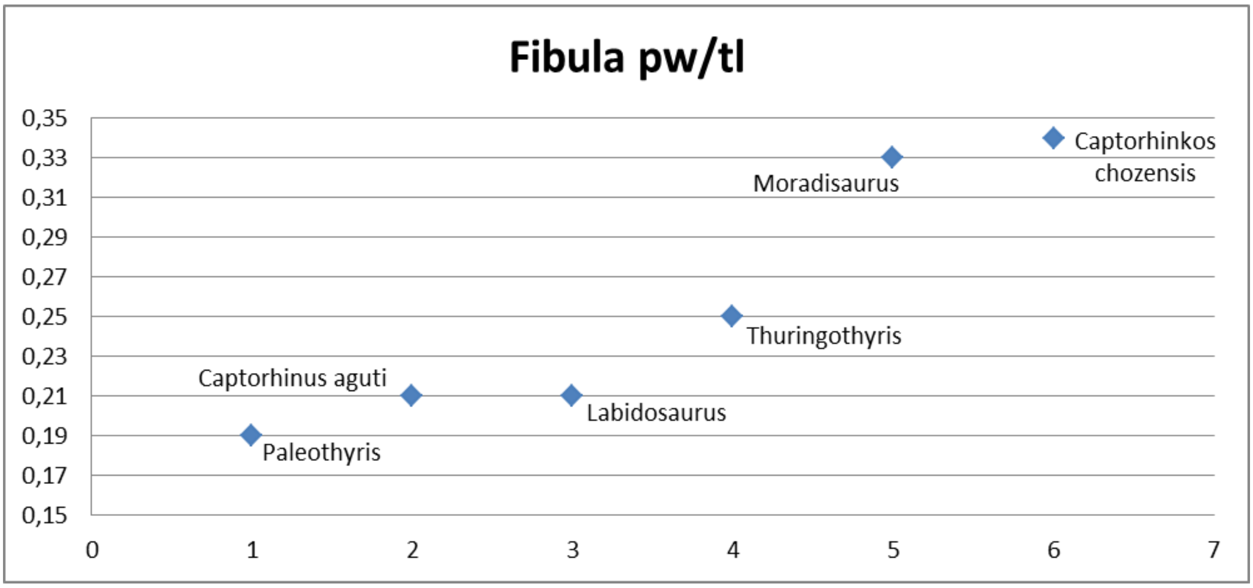
Fibula pw/tl
0,35
0,33
0,31
0,29
0,27
0,25
0,23
0,21
0,19
0,17
0,15
0
1
2
3
4
5
6
7
Captorhinkos chozensis
Moradisaurus
Thuringothyris
Captorhinus aguti
Labidosaurus
Paleothyris

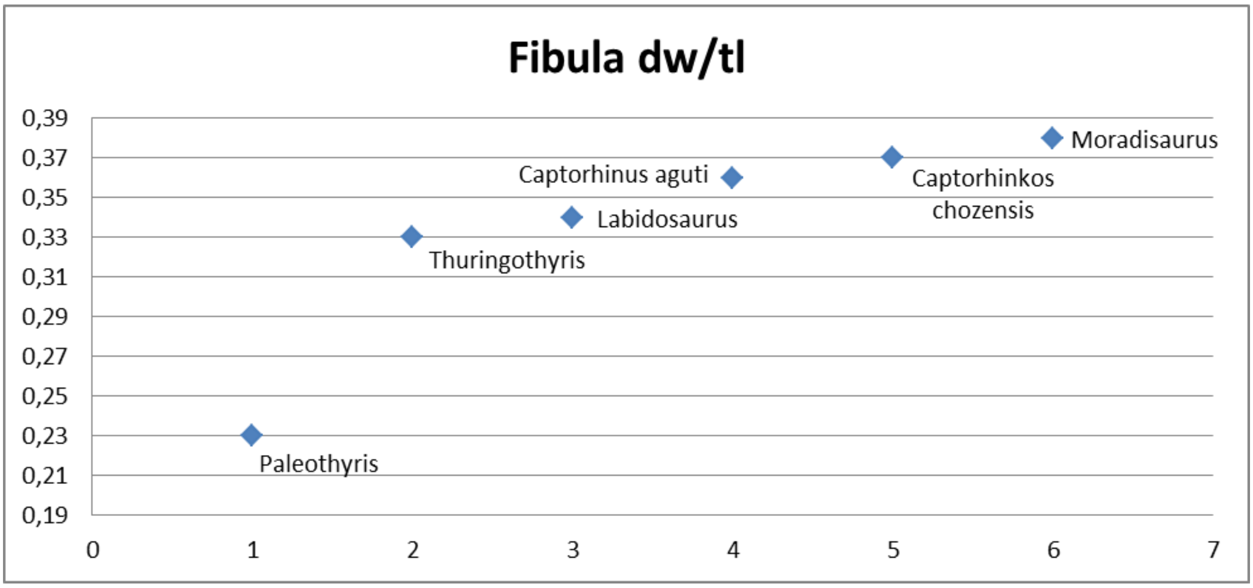
Fibula dw/tl
0,39
0,37
0,35
0,33
0,31
0,29
0,27
0,25
0,23
0,21
0,19
0
1
2
3
4
5
6
7
Moradisaurus
Captorhinus aguti
Captorhinkos chozensis
Labidosaurus
Thuringothyris
Paleothyris

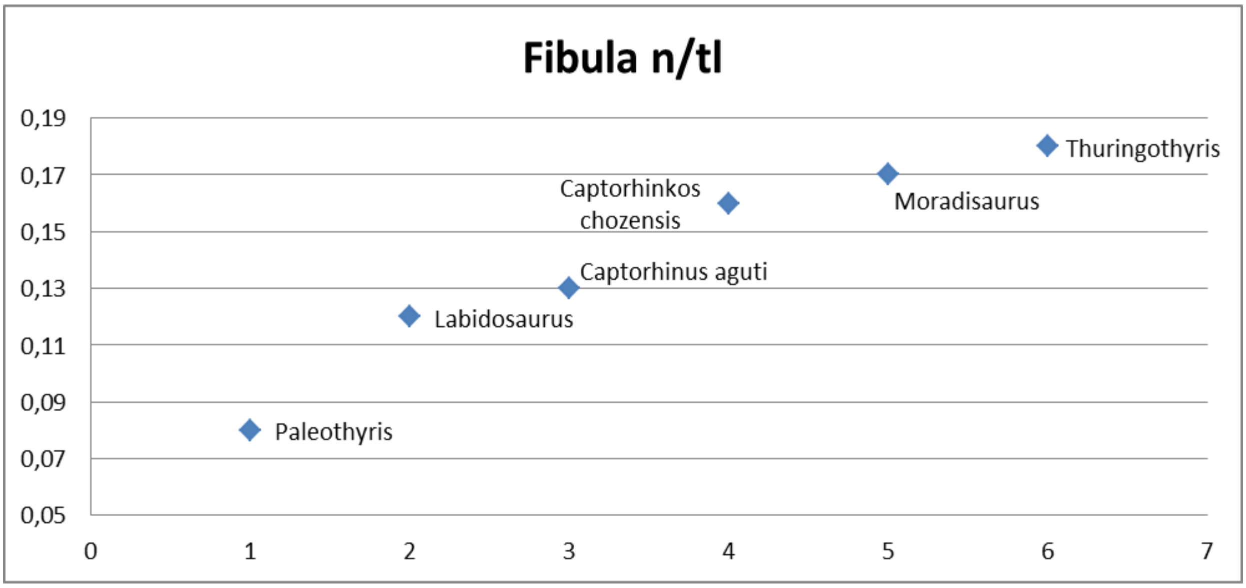
Fibula n/tl
0,19
0,17
0,15
0,13
0,11
0,09
0,07
0,05
0
1
2
3
4
5
6
7
Thuringothyris
Captorhinkos chozensis
Moradisaurus
Captorhinus aguti
Labidosaurus
Paleothyris

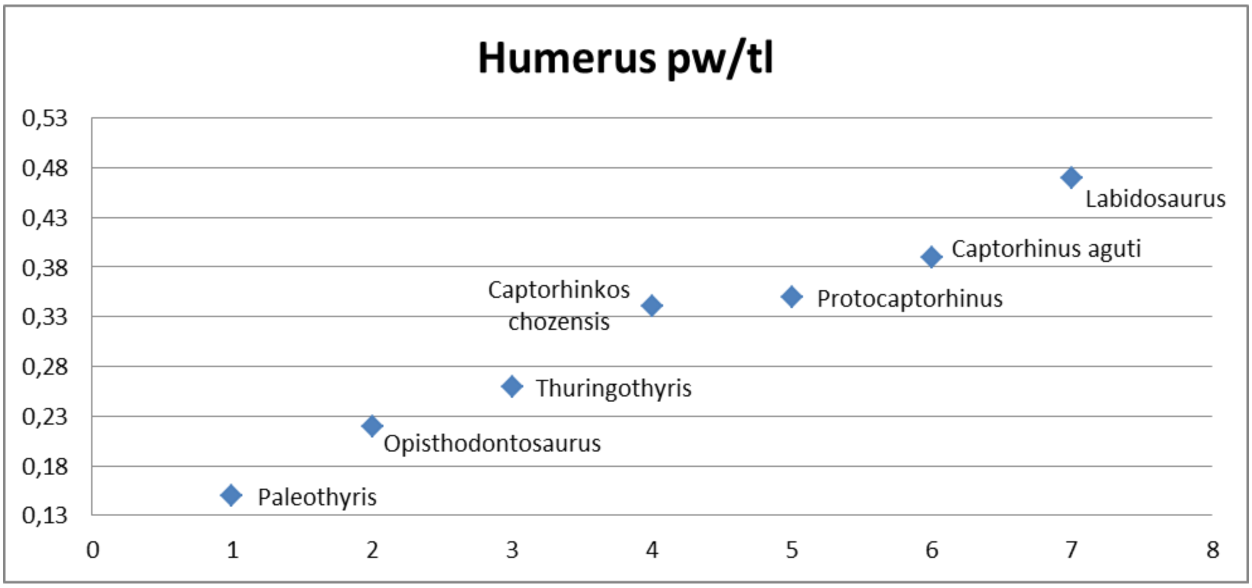
Humerus pw/tl
0,53
0,48
0,43
0,38
0,33
0,28
0,23
0,18
0,13
0
1
2
3
4
5
6
7
8
Labidosaurus
Captorhinus aguti
Captorhinkos chozensis
Protocaptorhinus
Thuringothyris
Opisthodontosaurus
Paleothyris

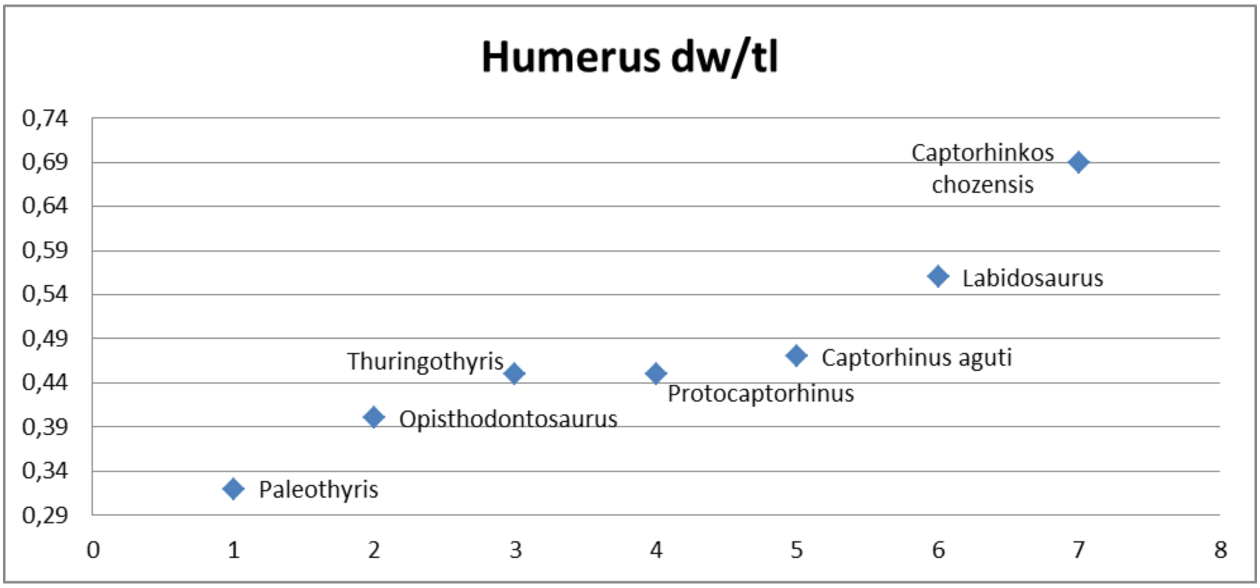
Humerus dw/tl
0,74
0,69
0,64
0,59
0,54
0,49
0,44
0,39
0,34
0,29
0
1
2
3
4
5
6
7
8
Captorhinkos chozensis
Labidosaurus
Thuringothyris
Captorhinus aguti
Protocaptorhinus
Opisthodontosaurus
Paleothyris

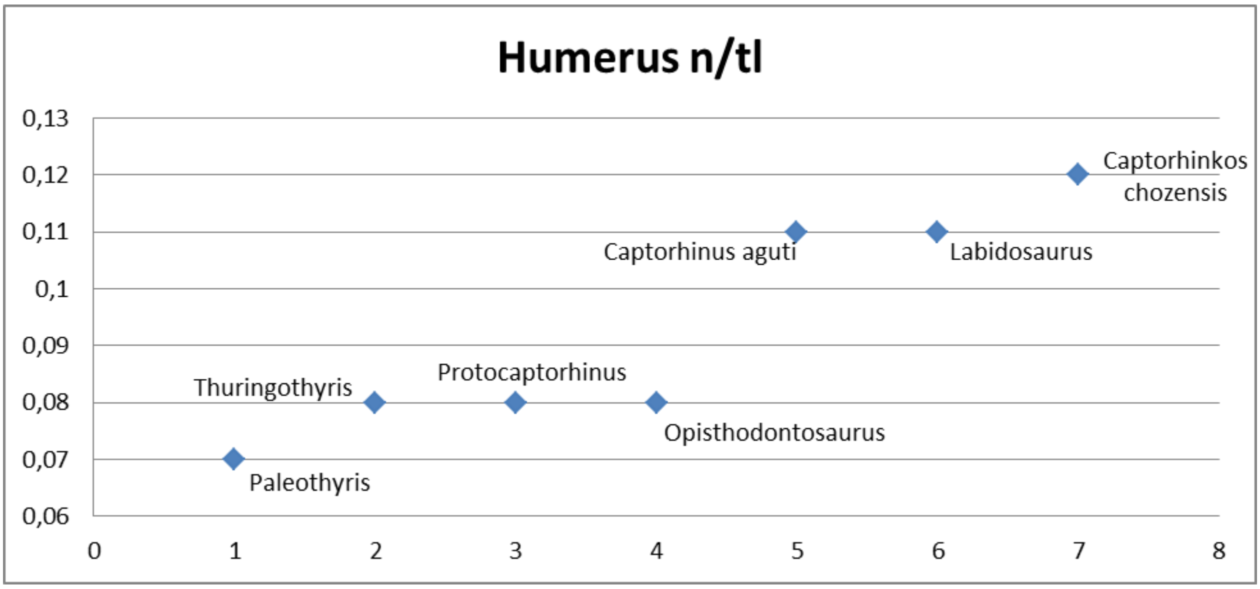
Humerus n/tl
0,13
0,12
0,11
0,1
0,09
0,08
0,07
0,06
0
1
2
3
4
5
6
7
8
Captorhinkos chozensis
Captorhinus aguti
Labidosaurus
Thuringothyris
Protocaptorhinus
Opisthodontosaurus
Paleothyris

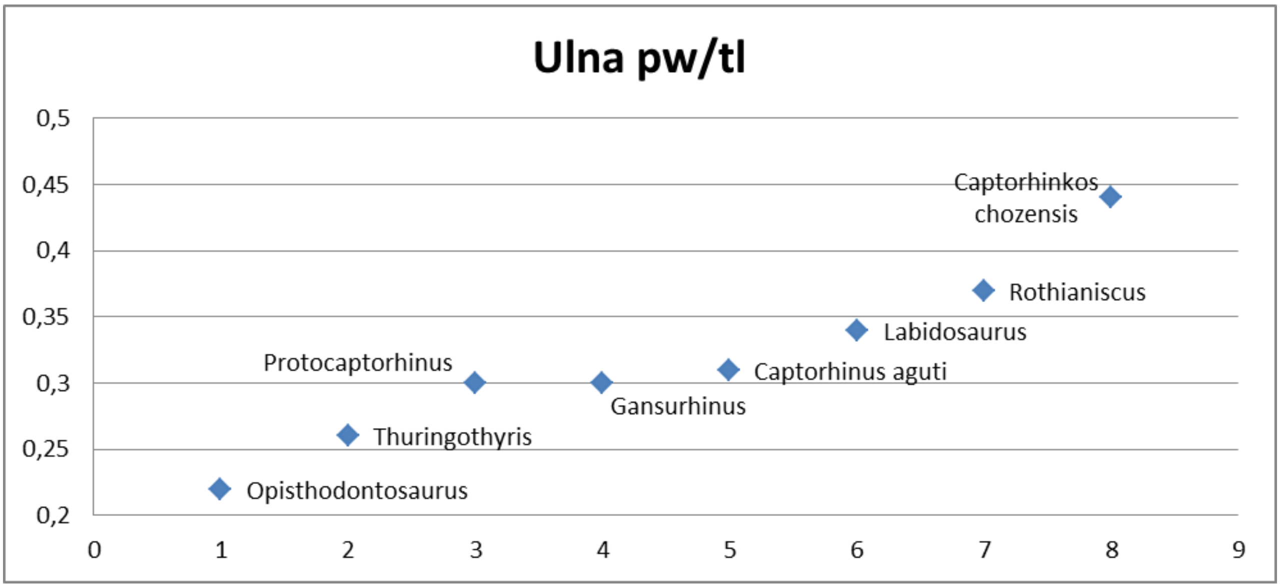
Ulna pw/tl
0,5
0,45
0,4
0,35
0,3
0,25
0,2
0
1
2
3
4
5
6
7
8
9
Captorhinkos chozensis
Rothianiscus
Labidosaurus
Captorhinus aguti
Protocaptorhinus
Gansurhinus
Thuringothyris
Opisthodontosaurus

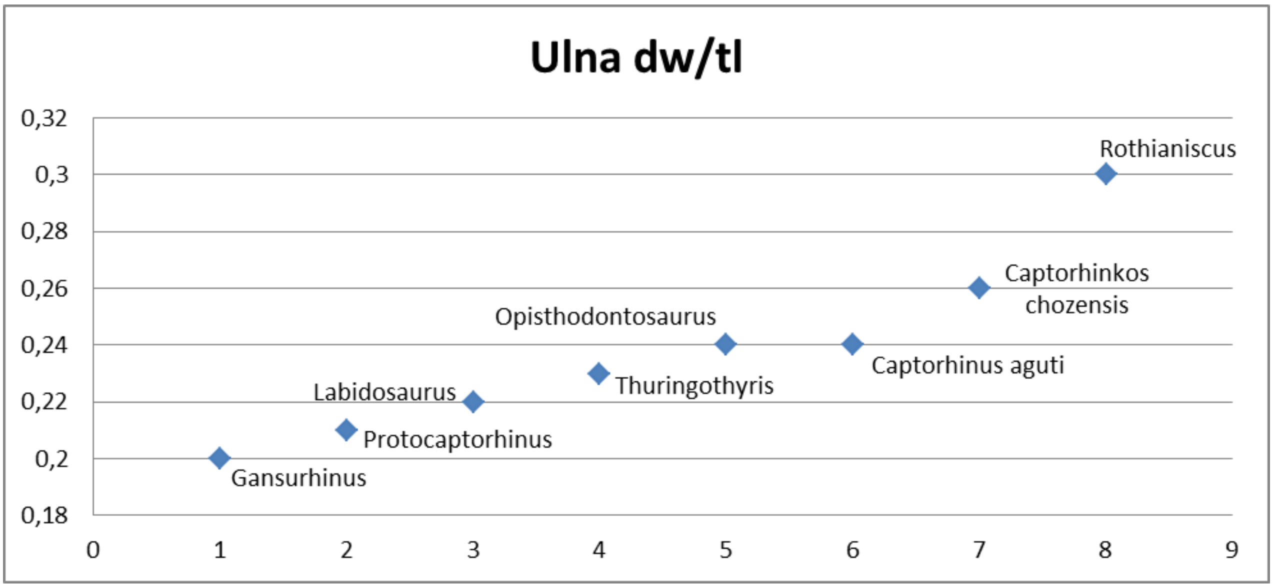
Ulna dw/tl
0,32
0,3
0,28
0,26
0,24
0,22
0,2
0,18
0
1
2
3
4
5
6
7
8
9
Rothianiscus
Captorhinkos chozensis
Opisthodontosaurus
Captorhinus aguti
Thuringothyris
Labidosaurus
Protocaptorhinus
Gansurhinus

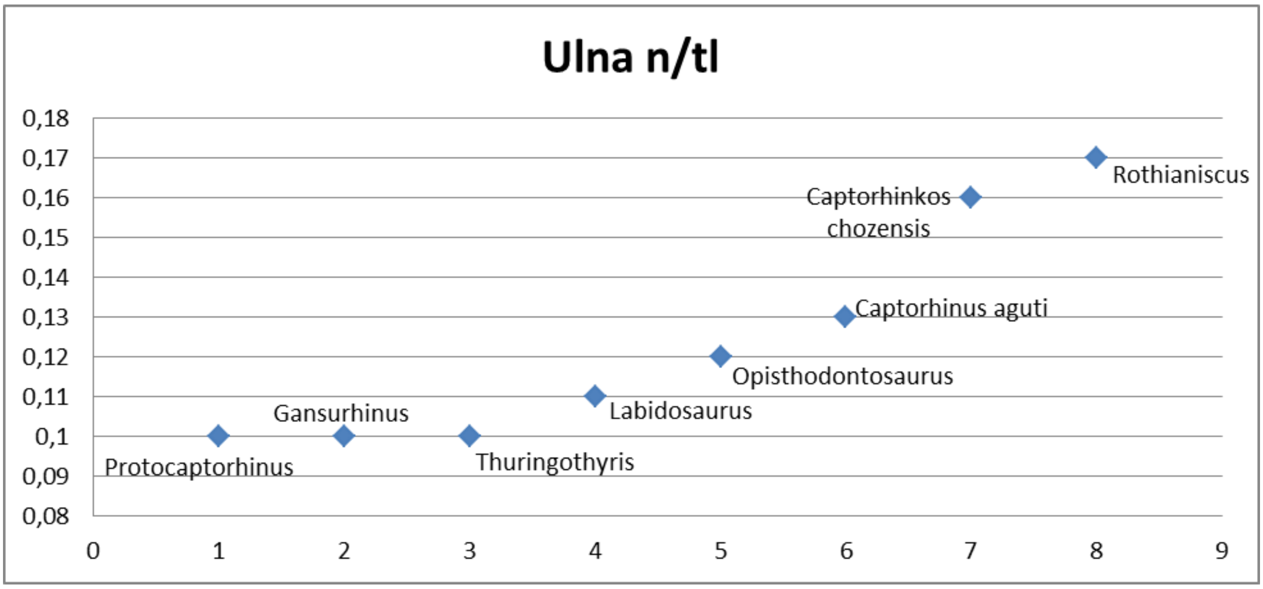
Ulna n/tl
0,18
0,17
0,16
0,15
0,14
0,13
0,12
0,11
0,1
0,09
0,08
0
1
2
3
4
5
6
7
8
9
Rothianiscus
Captorhinkos chozensis
Captorhinus aguti
Opisthodontosaurus
Labidosaurus
Gansurhinus
Protocaptorhinus
Thuringothyris

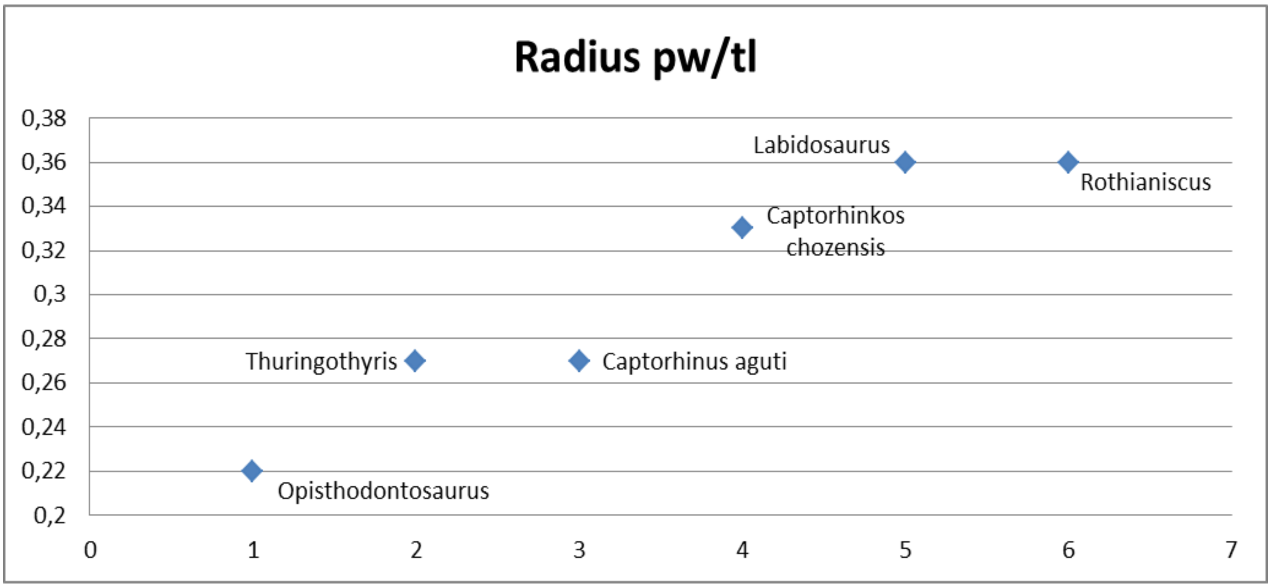
Radius pw/tl
0,38
0,36
0,34
0,32
0,3
0,28
0,26
0,24
0,22
0,2
0
1
2
3
4
5
6
7
Labidosaurus
Rothianiscus
Captorhinkos chozensis
Thuringothyris
Captorhinus aguti
Opisthodontosaurus

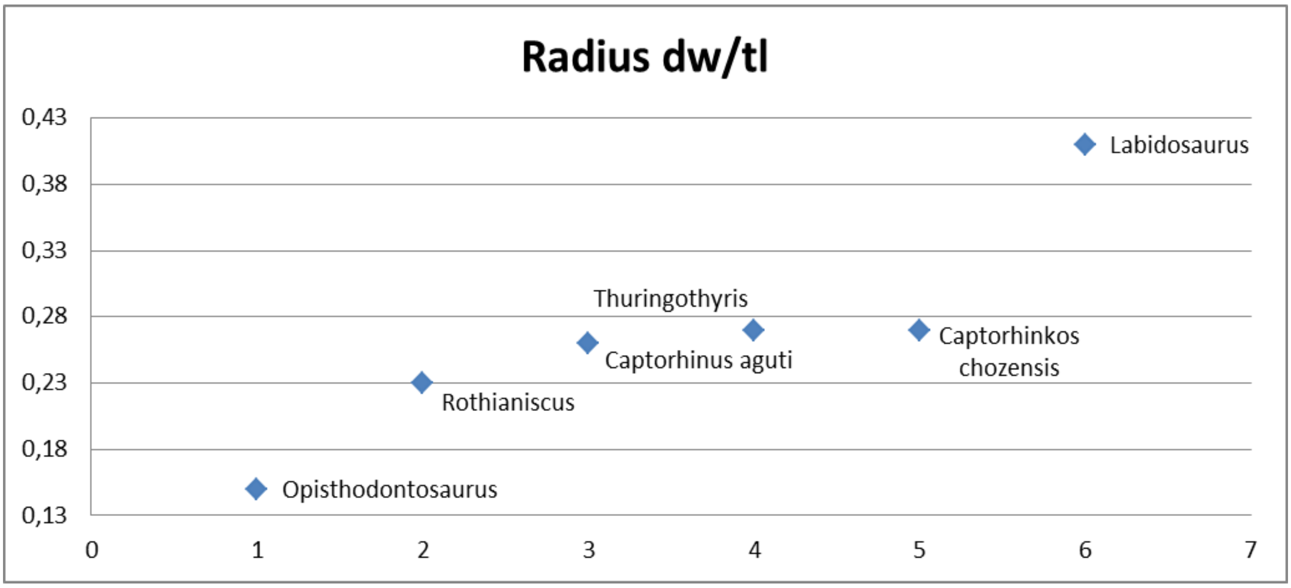
Radius dw/tl
0,43
0,38
0,33
0,28
0,23
0,18
0,13
0
1
2
3
4
5
6
7
Labidosaurus
Thuringothyris
Captorhinus aguti
Captorhinkos chozensis
Rothianiscus
Opisthodontosaurus

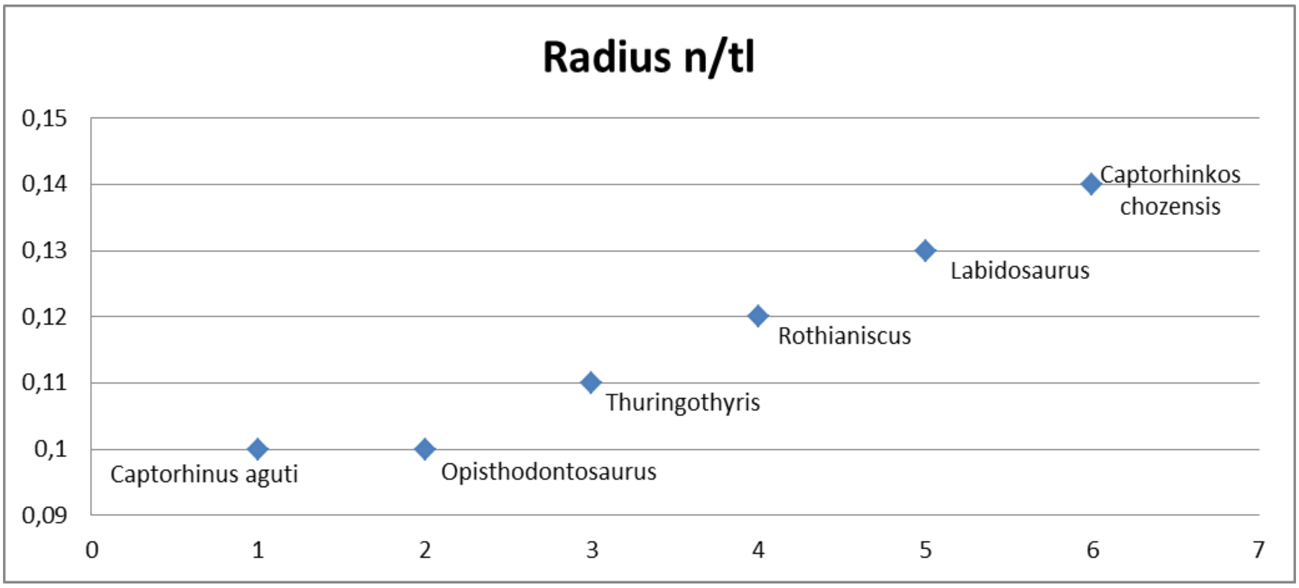
Radius n/tl
0,15
0,14
0,13
0,12
0,11
0,1
0,09
0
1
2
3
4
5
6
7
Captorhinkos chozensis
Labidosaurus
Rothianiscus
Thuringothyris
Captorhinus aguti
Opisthodontosaurus

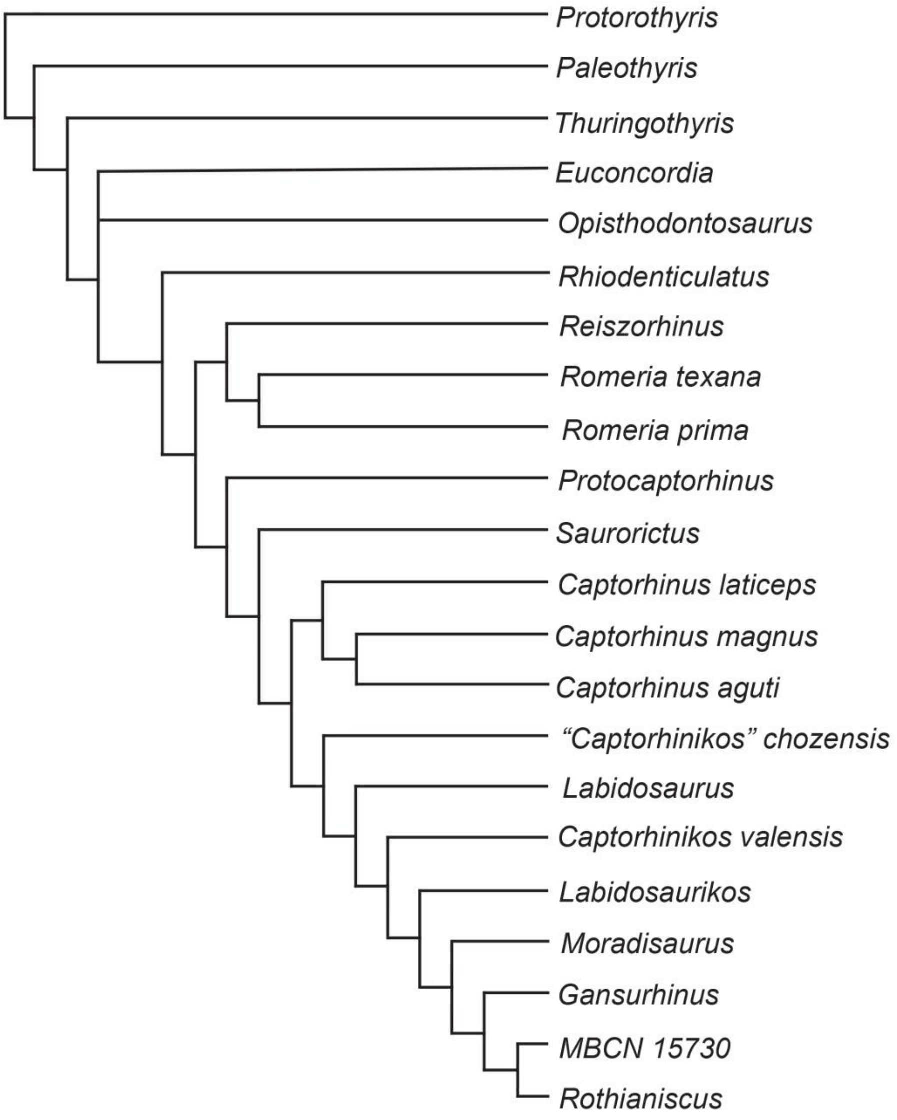

The strict consensus tree from the cladistic analysis performed on the original dataset of Liebrecht et al. (2016) showing a polytomy at the base of Captorhinidae.

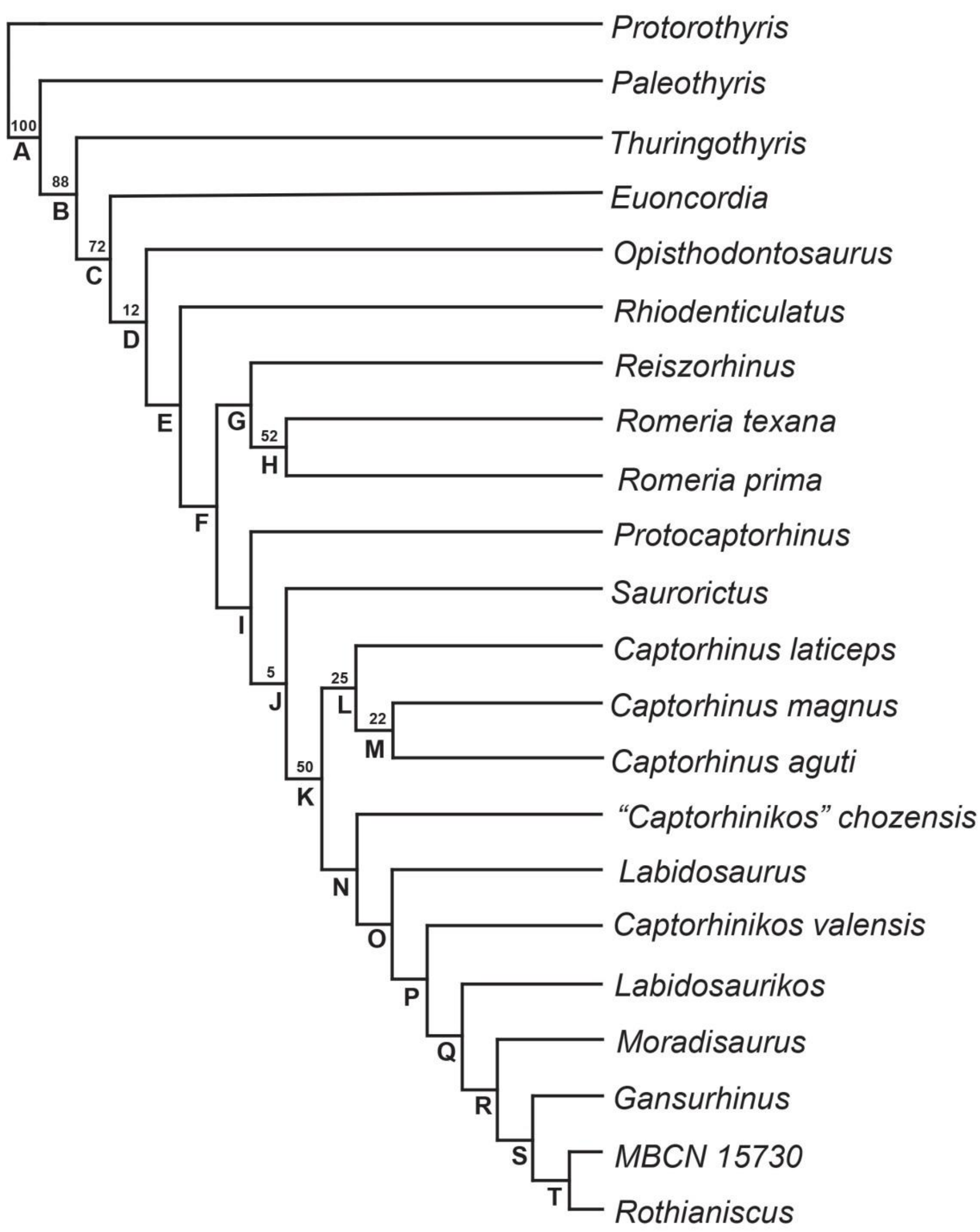

New fully resolved phylogeny of Captorhinidae. Numbers above nodes indicate the support obtained in TNT by the Symmetric Resampling with 10,000 replicates.
