# Supplementary material for: Discrete and continuous character-based disparity analyses converge to the same macroevolutionary signal: a case study from captorhinids
Source: Sci Rep. 2017 Dec 13;7:17531. doi: 10.1038/s41598-017-17757-5 (PMC5727480; doi:10.1038/s41598-017-17757-5)
Supplement: Supplementary file 2 — Appendix 2 [file 41598_2017_17757_MOESM2_ESM.pdf]

Supplementary information: Discrete and continuous character-based disparity analyses converge to the same macroevolutionary signal: a case study from captorhinids

Marco Romano[1,2,]* Neil Brocklehurst[1] and Jörg Fröbisch[1,3]

[1]Museum für Naturkunde, Leibniz-Institut für Evolutions- und Biodiversitätsforschung, Invalidenstr. 43, 10115 Berlin, Germany; [2]Dipartimento di Scienze della Terra, “Sapienza” Universita` di Roma, P.le A. Moro 5, 00185 Rome, Italy; [3]Institut für Biologie, Humboldt-Universität zu Berlin, Invalidenstr. 42, 10115 Berlin, Germany

Abstract

The relationship between diversity and disparity during the evolutionary history of a clade provides unique insights into evolutionary radiation and the biological response to bottlenecks and to extinctions. Here we present the first comprehensive comparison of diversity and disparity of captorhinids, a group of basal amniotes that is important for understanding the early evolution of high-fiber herbivory. A new fully resolved phylogeny is presented, obtained by the inclusion of 31 morphometric characters. The new dataset is used to calculate diversity and disparity through the evolutionary history of the clade, using both discrete and continuous characters. Captorhinids do not show a decoupling between diversity and disparity, and are characterized by a rather symmetric disparity distribution, with a peak in occupied morphospace at about the midpoint of the clade's evolutionary history (Kungurian). This peak represents a delayed adaptive radiation, identified by the first appearance of several high-fiber herbivores in the clade, along with numerous omnivorous taxa. The discrete characters and continuous morphometric characters indicate the same disparity trends. Therefore, we argue that in the absence of one of these two possible proxies, the disparity obtained from just one source can be considered robust and representative of a general disparity pattern.

Appendix 2. Data Matrix

nstates cont ;
xread
109 22

&[cont]

Protorothyris ? ? ? ? ? ? ? ? ?
? ? ? ? ? ? ? ? ? 0.33 0.38 0.56 0.49
0.35 0.32 0.26 0.40 0.20 0.30 0.18 0.18 0.10 0.31 0.83 0.62

Paleothyris 0.19 0.26 0.10 0.31 0.15 0.09 0.19 0.23 0.08
0.15 0.32 0.07 ? ? ? ? ? ? 0.34 0.39 0.58 0.49
0.37 0.31 0.25 0.40 0.22 0.29 0.14 0.17 0.12 0.26 0.94 1.05

Thuringothyris 0.30 0.34 0.16 ? ? ? 0.25 0.33 0.18
0.26 0.45 0.08 0.26 0.23 0.10 0.27 0.27 0.11 0.36 0.37 0.85 0.70
0.49 0.43 0.28 0.38 0.22 0.47 0.25 0.22 0.19 0.45 1.07 1.07

Concordia ? ? ? ? ? ? ? ? ?
? ? ? ? ? ? ? ? ? 0.40 0.31 0.92 ? ?
? 0.27 0.38 0.18 0.40 0.18 0.20 ? 0.30 0.95 1.00

Romeria prima ? ? ? ? ? ? ? ? ?
? ? ? ? ? ? ? ? ? 0.38 0.32 0.66 0.55
0.38 0.35 0.29 0.34 0.23 0.46 0.23 0.25 0.10 0.36 0.94 0.83

Romeria texana ? ? ? ? ? ? ? ? ?
? ? ? ? ? ? ? ? ? 0.40 0.35 0.68 0.51
0.35 0.33 0.22 0.36 0.27 0.40 0.22 0.16 0.12 0.46 0.87 1.25

Protocaptorhinus 0.39 ? 0.16 ? ? ? ? ? ?
0.35 0.45 0.08 0.30 0.21 0.10 ? ? ? 0.42 0.37 0.69 0.64
0.50 0.41 0.31 0.39 0.20 0.46 0.31 0.23 0.10 0.70 1.35 1.29

Rhiodentigculatus ? ? ? ? ? ? ? ? ?
? ? ? ? ? ? ? ? ? ? ? ? ? ?
? ? ? ? ? ? ? ? ? ? ?

Saurorictus ? ? ? ? ? ? ? ? ?
? ? ? ? ? ? ? ? ? 0.32 0.35 0.80 0.79
0.47 0.40 0.33 0.29 0.24 0.39 0.19 0.12 0.22 0.30 0.67 0.80

Captorhingus laticeps ? ? ? ? ? ? ? ? ?
? ? ? ? ? ? ? ? ? 0.39 0.36 0.83 0.67
0.42 0.37 0.26 0.35 0.23 0.34 0.21 0.20 0.19 0.39 1.06 1.12

Captorhinus aguti 0.34 0.37 0.13 0.49 0.25 0.12 0.21 0.36 0.13
0.39 0.47 0.11 0.31 0.24 0.13 0.27 0.26 0.10 0.43 0.33 0.84 0.62
0.38 0.32 0.32 0.36 0.22 0.38 0.20 0.17 0.17 0.42 1.31 1.23
Captorhinus magnus 0.40 0.41 0.18 ? ? ? ? ? ?
? ? ? ? ? ? ? ? ? ? ? ? ? ?
? ? ? ? ? ? ? ? ? ? ?

Labidosaurus 0.42 0.48 0.19 0.59 0.42 0.17 0.21 0.34 0.12
0.47 0.56 0.11 0.34 0.22 0.11 0.36 0.41 0.13 0.23 0.43 0.79 0.73
0.32 0.24 0.27 0.37 0.23 0.31 0.14 0.16 0.21 0.44 1.58 1.70

Labidosaurikos 0.47 ? ? ? ? ? ? ? ?
? ? ? ? ? ? ? ? ? 0.40 0.37 0.53 0.36
0.36 0.20 0.27 0.39 0.25 0.33 0.11 0.16 0.19 0.62 1.92 1.99

Moradisaurus ? 0.47 0.17 0.48 0.27 0.17 0.33 0.38 0.17
? ? ? ? ? ? ? ? ? ? ? ? ? ?
? ? ? ? ? ? ? ? ? ? ?

Rothianiscus 0.39 0.43 0.09 ? ? ? ? ? ?
? ? ? 0.37 0.30 0.17 0.36 0.23 0.12 ? ? ? ? ?
? ? ? ? ? ? ? ? ? ? ?

Captorhinikos valensis ? ? ? ? ? ? ? ? ?
? ? ? ? ? ? ? ? ? ? ? ? ? ?
? ? ? ? ? ? ? ? ? ? ?

Gansurhinus ? ? ? ? ? ? ? ? ?
? ? ? 0.30 0.20 0.10 ? ? ? ? ? ? ? ?
? ? ? ? ? ? ? ? ? ? ?

Captorhinkos chozensis 0.42 0.45 0.21 0.48 0.44 0.16 0.34 0.37 0.16
0.34 0.69 0.12 0.44 0.26 0.16 0.33 0.27 0.14 0.37 0.42 0.91 0.77
0.50 0.45 ? ? 0.34 ? 0.21 0.19 ? 0.47 ? ?

Reiszorhinus ? ? ? ? ? ? ? ? ?
? ? ? ? ? ? ? ? ? 0.39 0.40 ? ? ?
? 0.25 0.38 0.23 ? ? ? ? 0.32 1.09 1.27

Opisthodontosaurus 0.30 0.31 0.13 ? ? ? ? ? ?
0.22 0.40 0.08 0.22 0.24 0.12 0.22 0.15 0.10 ? ? ? ? ?
? ? ? ? ? ? ? ? 0.34 1.17 1.16

MBCN ? ? ? ? ? ? ? ? ? ?
? ? ? ? ? ? ? ? ? ? ? ? ? ?
? ? ? ? ? ? ? ? ? ?

&[numeric]

Protorothyris 00000 00000 00000 00001 00010 01000 00100 00000 00000
10000 00000 00000 00000 0111? 011??

Paleothyris 00000 00000 00000 01001 00000 00200 00000 00000 00000
00?00 00000 00000 0???0 01111 01111

Thuringothyris 00000 10100 00100 11000 01000 01100 00100 00000 00010
10000 00000 ??000 ????0 011?? 01011

Concordia 01001 10000 00110 11000 10??0 01100 00010 00?00 00010
00?00 0000? 00000 0???0 ????? ?????

Romeria prima 11110 10000 00100 00001 11010 000?0 00010 01?0? ?0?10
10000 10?0? 10000 0???0 ?0?0? 1?0??

Romeria texana 11110 10000 00??0 01001 11010 011?0 00010 0100? 00010
?0?0? 10?00 10000 0???0 ????? ?????

Protocaptorhinus 11110 10000 00110 00000 110?0 00101 00110 1???1 ?0010
?0??0 1000? ?0000 0???1 10??1 020??

Rhiodenticulatus 11010 10000 00110 11000 10100 011?? ?0110 1?101 00010
1000? 1?000 10000 01??? 1?01? 020??

Saurorictus ?1111 10000 001?0 00000 00100 0000? ??110 ????? ???1?
????? ?0??? 100?? ????? ????? ?????

Captorhinus laticeps 11111 10001 00110 00001 10100 11101 00110 21102 00111
11000 10000 11001 01002 10000 02000

Captorhinus aguti 11111 11012 01110 00000 10100 11101 00110 21102 00111
11000 10000 11001 01002 10000 02000

Captorhinus magnus 11111 10002 00110 00000 10100 11101 00110 21102 001?1
11000 10000 11001 010?2 100?? 120??

Labidosaurus 11111 10101 00101 01110 10100 11111 01110 21101 00112
11001 11110 11111 11111 10000 12001

Labidosaurikos 11111 22133 12101 01110 10101 10111 11111 21111 11112
11101 11110 2?111 10111 ????? ?????

Moradisaurus 1?110 22033 011?0 1??1? ????? 2?11? 1???1 11111 11122
11111 ?1101 21111 10111 20??? 1?00?

Rothianiscus 1?110 31123 12??1 ????0 ????1 2?11? 1???? 1111? 1212?
11??? ????? 2?111 ????1 200?? 0?0??

Captorhiniko valensis ????? 12123 02??? ????? ????? ????? ????? ????? 000??
????? ????? 2???? ????? 20??? ?????

Gansurhinus 1??1? 32123 00??? ????? ????? ????? ????? ????? ?????
?1??? ????1 ????? ????? 200?? ?2???

Captorhinkos chozensis 11111 11111 011?0 000?1 ????? 1?10? ????? ???01 001??
1???? 1??1? 20101 1???1 1?000 110?0

Reiszorhinus 11110 10000 00100 10000 10000 01100 00010 1???? ?????
????? 1000? 10010 ?11?0 ????? ?????

Opisthodontosaurus ??011 30003 001?? ???01 110?0 1110? ????? ?0?02 00010
010?? 1???? 01000 11010 1?1?? 121??

MBCN ????? 31?13 ?2??? ????0 ????? ????? ????? ???1? ?????
????? ????? ????? ????? ????? ?????
