# Supplementary material for: Discrete and continuous character-based disparity analyses converge to the same macroevolutionary signal: a case study from captorhinids
Source: Sci Rep. 2017 Dec 13;7:17531. doi: 10.1038/s41598-017-17757-5 (PMC5727480; doi:10.1038/s41598-017-17757-5)
Supplement: Supplementary file 3 — Appendix 3 [file 41598_2017_17757_MOESM3_ESM.pdf]

Supplementary information: Discrete and continuous character-based disparity analyses converge to the same macroevolutionary signal: a case study from captorhinids

Marco Romano[1,2,]* Neil Brocklehurst[1] and Jörg Fröbisch[1,3]

[1]Museum für Naturkunde, Leibniz-Institut für Evolutions- und Biodiversitätsforschung, Invalidenstr. 43, 10115 Berlin, Germany; [2]Dipartimento di Scienze della Terra, "Sapienza" Universita` di Roma, P.le A. Moro 5, 00185 Rome, Italy; [3]Institut für Biologie, Humboldt-Universität zu Berlin, Invalidenstr. 42, 10115 Berlin, Germany

Abstract

The relationship between diversity and disparity during the evolutionary history of a clade provides unique insights into evolutionary radiation and the biological response to bottlenecks and to extinctions. Here we present the first comprehensive comparison of diversity and disparity of captorhinids, a group of basal amniotes that is important for understanding the early evolution of high-fiber herbivory. A new fully resolved phylogeny is presented, obtained by the inclusion of 31 morphometric characters. The new dataset is used to calculate diversity and disparity through the evolutionary history of the clade, using both discrete and continuous characters. Captorhinids do not show a decoupling between diversity and disparity, and are characterized by a rather symmetric disparity distribution, with a peak in occupied morphospace at about the midpoint of the clade's evolutionary history (Kungurian). This peak represents a delayed adaptive radiation, identified by the first appearance of several high-fiber herbivores in the clade, along with numerous omnivorous taxa. The discrete characters and continuous morphometric characters indicate the same disparity trends. Therefore, we argue that in the absence of one of these two possible proxies, the disparity obtained from just one source can be considered robust and representative of a general disparity pattern.

Appendix 3

Disparity Metric	Continuous Characters		Discrete characters	
	Without Ghost Lineages	With Ghost Lineages	Without Ghost Lineages	With Ghost Lineages
Sum of Variance	0.4713645	0.6070098	0.5683029	0.6555773
Sum of Ranges	0.4333486	0.4628456	0.487942	0.50818

Table 1: The centre of mass of each disparity curve. Values closer to 0 indicate a “bottom heavy” clade (i.e. disparity concentrated earlier), while values closer to 1 indicate a “top heavy” clade (i.e. disparity concentrated later)

Correlation tested	Pearson’s R	P-value
PC 1 vs PCo 1	0.12282887	0.616402844
PC 2 vs PCo 2	0.110357186	0.652885755
PC 3 vs PCo 3	0.293639227	0.222399376
PC 4 vs PCo 4	0.042718883	0.862144987
PC 5 vs PCo 5	-0.045020175	0.854791773
PC 6 vs PCo 6	-0.234179931	0.334556416
PC 7 vs PCo 7	0.313335475	0.19146291
PC 8 vs PCo 8	-0.062657834	0.798856495
PC 9 vs PCo 9	0.490249667	0.033093979
PC 10 vs PCo 10	-0.066251158	0.787565958
PC 11 vs PCo 11	0.015348376	0.950273463
PC 12 vs PCo 12	-0.13242284	0.588906907
PC 13 vs PCo 13	0.226706448	0.350646826
PC 14 vs PCo 14	0.301105452	0.210312585

Table 2: Results of correlation tests of the respective Principal Component (PC) and Principal Coordinate (PCo) scores of each taxon, after correcting for the phylogenetic non-independence of taxa using independent contrasts.
